# Supplementary material for: Effective AAV-mediated gene replacement therapy in retinal organoids modeling AIPL1-associated LCA4
Source: Mol Ther Nucleic Acids. 2024 Feb 13;35(1):102148. doi: 10.1016/j.omtn.2024.102148 (PMC10910061; doi:10.1016/j.omtn.2024.102148)
Supplement: Document S1. Figures S1–S6 and Tables S1 and S2 [file mmc1.pdf]

OMTN, Volume *35*

Supplemental information

Effective AAV-mediated gene replacement therapy in retinal organoids modeling AIPL1-associated LCA4

Hali Sai, Bethany Ollington, Farah O. Rezek, Niuzheng Chai, Amelia Lane, Tassos Georgiadis, James Bainbridge, Michel Michaelides, Almudena Sacristan-Reviriego, Pedro R.L. Perdigão, Amy Leung, and Jacqueline van der Spuy

SUPPLEMENTAL INFORMATION

SUPPLEMENTAL TABLES

Table S1. Primary and secondary antibodies used in immunofluorescence (IF) and western blot (WB) analysis.

Antibody	Species	Fluorophore conjugate	Supplier	Cat number	Dilution
AIPL1	Rabbit	-	Produced in-house	N/A	1:250 (IF)
AIPL1	Rabbit	-	V. Ramamurthy lab	N/A	1:1000 (WB)
Cone arrestin	Mouse	-	Millipore	MABN2636	1:250 (IF)
GAPDH	Mouse	-	ProteinTech	60004-1-Ig	1:10,000 (WB)
PDE6A	Rabbit	-	ProteinTech	21200-1-AP	1:1000 (IF)<br>1:3000 (WB)
PDE6B	Rabbit	-	Thermo Fisher Scientific	PA1-722	1:500 (IF)
Rhodopsin	Mouse	-	Millipore	MABN15	1:1000 (IF)
α-mouse IgG	Donkey	AF488	Thermo Fisher Scientific	A-21202	1:1000 (IF)
α-mouse IgG	Donkey	AF555	Thermo Fisher Scientific	A-32773	1:1000 (IF)
α-mouse IgG	Donkey	AF647	Thermo Fisher Scientific	A-31571	1:1000 (IF)
α-rabbit IgG	Donkey	AF488	Thermo Fisher Scientific	A-21206	1:1000 (IF)
α-rabbit IgG	Donkey	AF555	Thermo Fisher Scientific	A-31572	1:1000 (IF)
α-rabbit IgG	Donkey	AF647	Thermo Fisher Scientific	A-31573	1:1000 (IF)
α-mouse IgG	Goat	HRP	Thermo Fisher Scientific	31430	1:10,000 (WB)
α-rabbit IgG	Goat	HRP	Thermo Fisher Scientific	31461	1:10,000 (WB)

Phalloidin	-	AF488	Invitrogen / Molecular probes	A12379	5 U/mL (IF)

Table S2. Primer sequences for RT-PCR analysis of AIPL1-associated markers.

Marker	Forward	Reverse	Expected product size (bp)
AIPL1	ACCGGATCCCGA GTGATCTT	CGATGATGATGTG CATGGGC	66
CRX	TTTGCCAAGACC CAGTACC	GTTCTTGAACCAA ACCTGAAC	96
GRK1	GAAGCTGAACAA GAAGCGG	GACACGATGAACC TGCTGT	99
PDE6A	TAACGTCCCCAA CACAGAGG	CCACCACATCCTT CCCATTC	116
PDE6B	GACGTGTGGTCT GTGCTGAT	CTTGCCGTGGAGG ATGTAGTC	111
PDE6C	GTCACTAAGAAC CTGCTGGCAACC	AAAGACCTCTTCA TCCTGTTTGG	117
PDE6G	AAGCAGCGACAG ACCAGG	TGTGATGTCTGTT CCCAGGC	105
PDE6H	GAGGCAGACTCG CCAATTC	GTGGCTGAATGCC TCCCA	130

SUPPLEMENTAL FIGURES

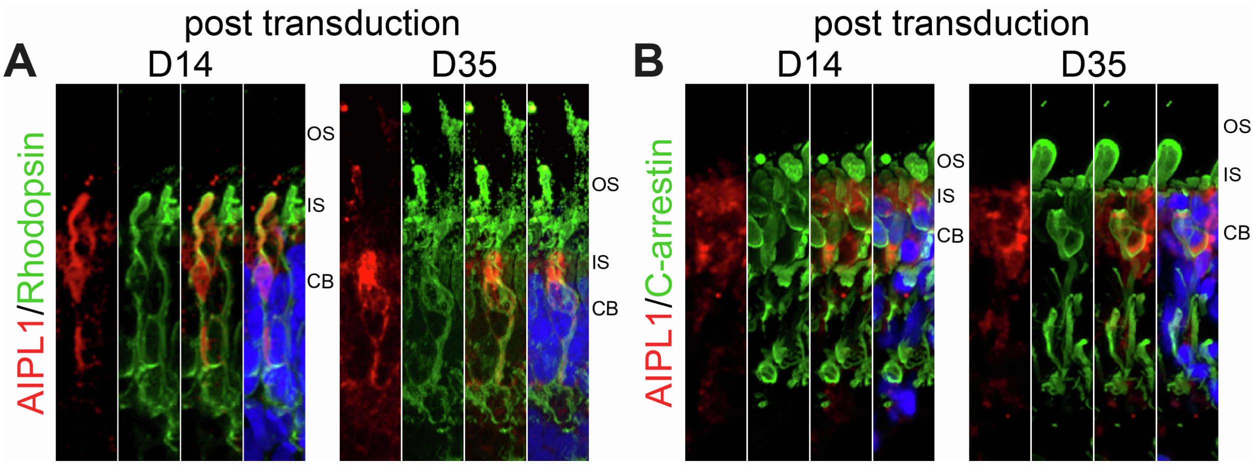

Figure S1: AAV transduction of rod and cone photoreceptors

(A) Expression of AIPL1 (red) and rhodopsin (green) and (B) AIPL1 (red) and cone arrestin (green) in LCA4 patient-derived (LCA4) ROs transduced with AAV7m8.*hRKp.AIPL1* at day 196 of differentiation and analysed 14 days (D14) and 35 days (D35) post transduction.

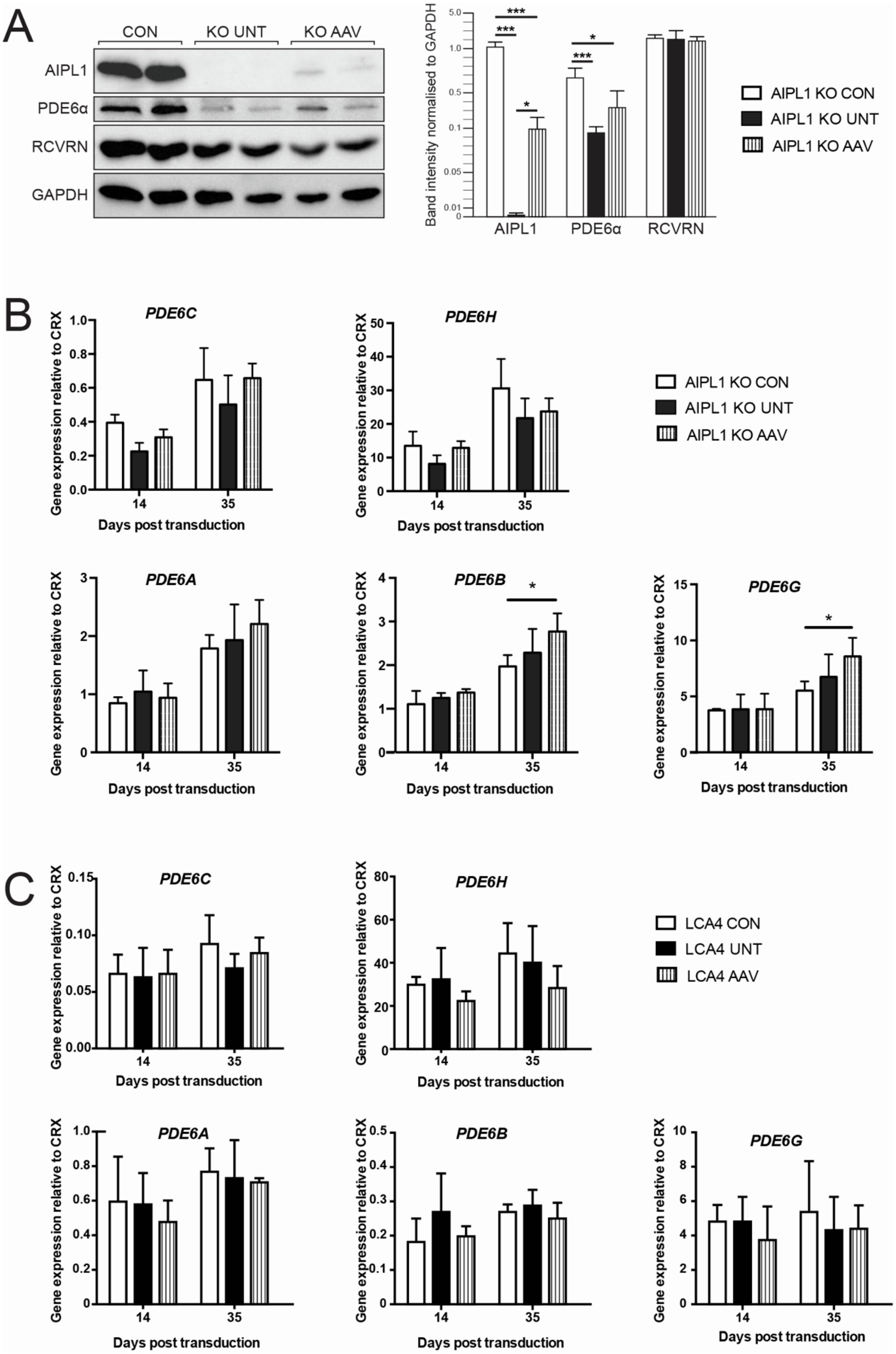
A
CON
KO UNT
KO AAV
AIPL1
PDE6α
RCVRN
GAPDH
Band intensity normalised to GAPDH
AIPL1
PDE6α
RCVRN
AIPL1 KO CON
AIPL1 KO UNT
AIPL1 KO AAV
B
PDE6C
PDE6H
PDE6A
PDE6B
PDE6G
Gene expression relative to CRX
Days post transduction
C
LCA4 CON
LCA4 UNT
LCA4 AAV

Figure S2: Rescue of PDE6 is post-transcriptional

(A) Key expression of LCA4 related proteins following AAV7m8.*hRKp.AIPL1* treatment in the AIPL1 KO model. Western blot of 3-6 pooled ROs per lane from isogenic control (CON), AIPL1 KO untreated (KO UNT) and AIPL1 KO AAV treated (KO AAV) ROs probed for AIPL1, PDE6α, Recoverin (RCVRN) and GAPDH as a loading control, and densitometry calculated relative to GAPDH (n = 2 independent experiments). Statistical significance determined by one-way ANOVA where *, **, *** denote a p value ≤0.05, ≤0.01 and ≤0.005 respectively. Total RNA was isolated from AIPL1 KO ROs (B) and LCA4 patient-derived ROs (C) treated with AAV7m8.*hRKp.AIPL1* at day 196 of differentiation and analysed 14 days (2 weeks) and 35 days (5 weeks) post transduction. The expression of *PDE6C*, *PDE6H*, *PDE6A*, *PDE6B* and *PDE6G* in isogenic control (CON), untreated (UNT) and AAV treated (AAV) ROs was assessed by qPCR relative to the expression of *CRX*. N = 3 individual ROs per sample (with n = 3 technical replicates per RO). Statistical significance determined by one-way ANOVA, where *, **, *** denotes p<0.05, 0.01 and 0.005 respectively.

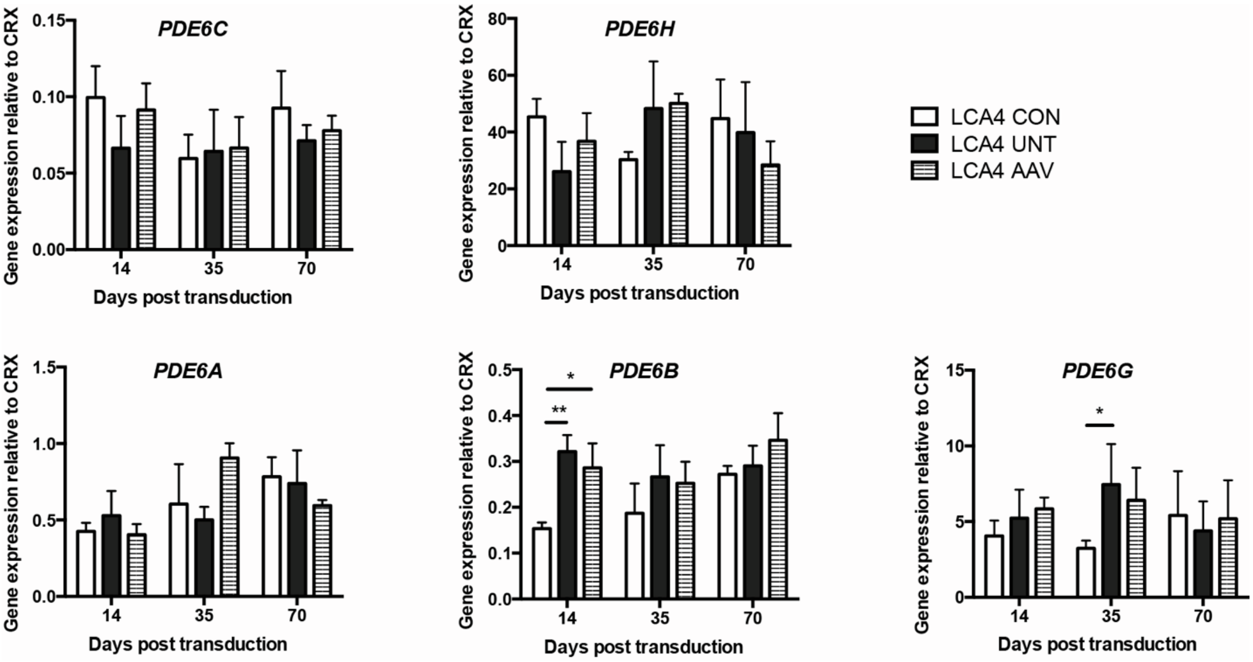

Figure S3: PDE6 transcript levels are unchanged several weeks after AAV treatment

Total RNA was isolated from LCA4 patient-derived ROs treated with AAV7m8.*hRKp.AIPL1* at day 161 of differentiation and analysed 14 days (2 weeks), 35 days (5 weeks) and 70 days (10 weeks) post transduction. The expression of *PDE6C*, *PDE6H, PDE6A*, *PDE6B*, and *PDE6G* in isogenic control (CON), untreated (UNT) and AAV treated (AAV) ROs was assessed by qPCR relative to the expression of *CRX*. N = 3 individual ROs per sample (with n = 3 technical replicates per RO). Statistical significance determined by one-way ANOVA, where *, **, *** denotes p<0.05, 0.01 and 0.005 respectively.

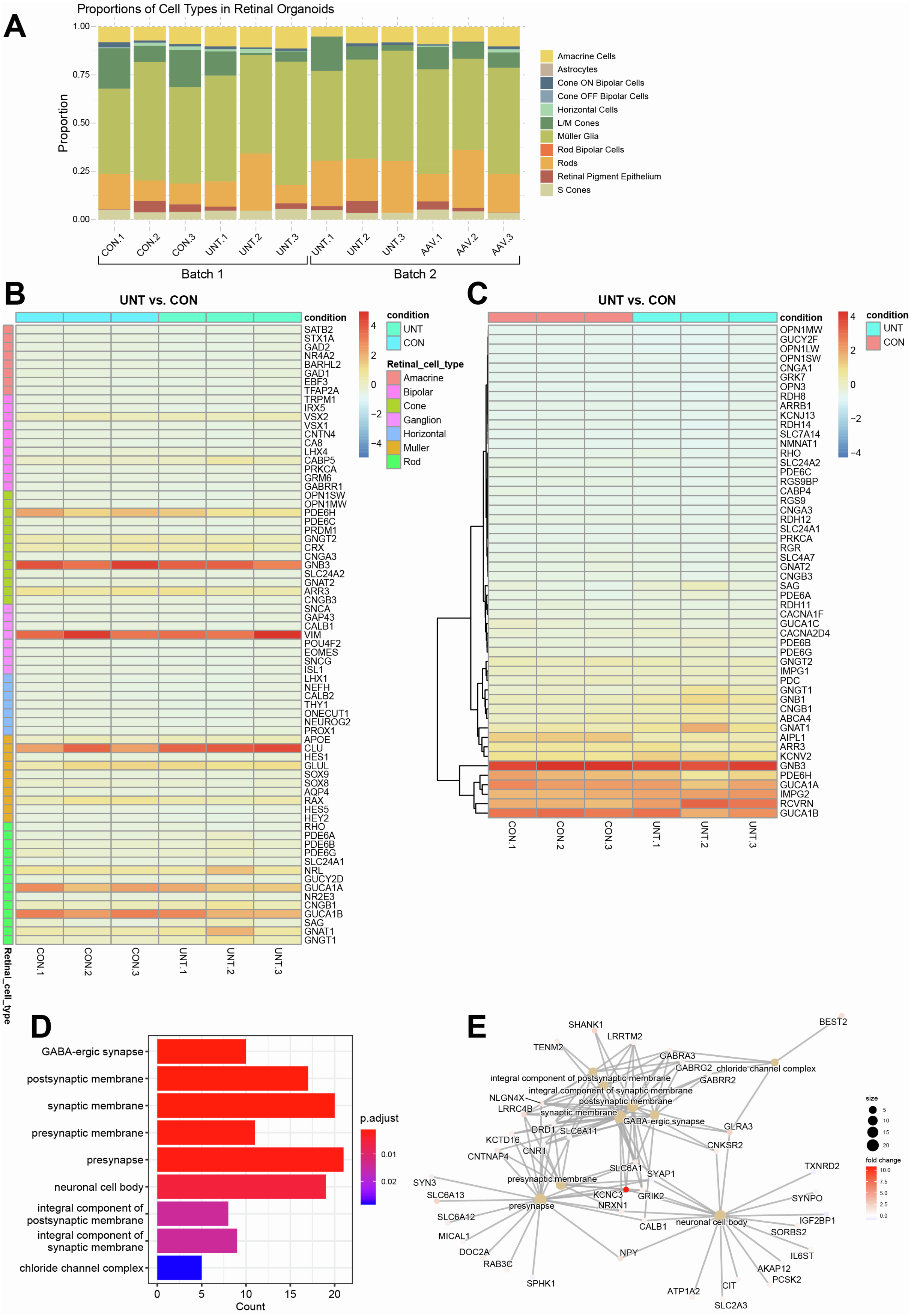
A
Proportions of Cell Types in Retinal Organoids
Proportion
1.00
0.75
0.50
0.25
0.00
CON.1
CON.2
CON.3
UNT.1
UNT.2
UNT.3
UNT.1
UNT.2
UNT.3
AAV.1
AAV.2
AAV.3
Batch 1
Batch 2
Amacrine Cells
Astrocytes
Cone ON Bipolar Cells
Cone OFF Bipolar Cells
Horizontal Cells
L/M Cones
Müller Glia
Rod Bipolar Cells
Rods
Retinal Pigment Epithelium
S Cones
B
UNT vs. CON
condition
SATB2 STX1A GAD2 NR4A2 BARHL2 GAD1 EBF3 TFAP2A TRPM1 IRX5 VSX2 VSX1 CNTN4 CA8 LHX4 CABP5 PRKCA GRM6 GABRR1 OPN1SW OPN1MW PDE6H PDE6C PRDM1 GNGT2 CRX CNGA3 GNB3 SLC24A2 GNAT2 ARR3 CNGB3 SNCA GAP43 CALB1 VIM POU4F2 EOMES SNCG ISL1 LHX1 NEFH CALB2 THY1 ONECUT1 NEUROG2 PROX1 APOE CLU HES1 GLUL SOX9 SOX8 AQP4 RAX HES5 HEY2 RHO PDE6A PDE6B PDE6G SLC24A1 NRL GUCY2D GUCA1A NR2E3 CNGB1 GUCA1B SAG GNAT1 GNGT1
CON.1 CON.2 CON.3 UNT.1 UNT.2 UNT.3
Retinal_cell_type
4 2 0 −2 −4
condition
UNT
CON
Retinal_cell_type
Amacrine
Bipolar
Cone
Ganglion
Horizontal
Muller
Rod
C
UNT vs. CON
condition
OPN1MW GUCY2F OPN1LW OPN1SW CNGA1 GRK7 OPN3 RDH8 ARRB1 KCNJ13 RDH14 SLC7A14 NMNAT1 RHO SLC24A2 PDE6C RGS9BP CABP4 RGS9 CNGA3 RDH12 SLC24A1 PRKCA RGR SLC4A7 GNAT2 CNGB3 SAG PDE6A RDH11 CACNA1F GUCA1C CACNA2D4 PDE6B PDE6G GNGT2 IMPG1 PDC GNGT1 GNB1 CNGB1 ABCA4 GNAT1 AIPL1 ARR3 KCNV2 GNB3 PDE6H GUCA1A IMPG2 RCVRN GUCA1B
CON.1 CON.2 CON.3 UNT.1 UNT.2 UNT.3
4 2 0 −2 −4
condition
UNT
CON
D
GABA-ergic synapse
postsynaptic membrane
synaptic membrane
presynaptic membrane
presynapse
neuronal cell body
integral component of postsynaptic membrane
integral component of synaptic membrane
chloride channel complex
0 5 10 15 20
Count
p.adjust
0.01
0.02
E
SHANK1 LRRTM2 TENM2 GABRA3 GABRG2 GABRR2 BEST2 chloride channel complex
integral component of postsynaptic membrane
integral component of synaptic membrane
NLGN4X LRRC4B synaptic membrane postsynaptic membrane GABA-ergic synapse GLRA3
DRD1 SLC6A11 KCTD16 CNR1 CNTNAP4 CNKSR2 TXNRD2
SLC6A1 SYAP1 SYN3 presynaptic membrane SLC6A13 KCNC3 GRIK2 SYNPO presynapse NRXN1
SLC6A12 CALB1 neuronal cell body IGF2BP1 MICAL1 SORBS2 DOC2A NPY IL6ST RAB3C AKAP12 PCSK2 SPHK1 ATP1A2 CIT SLC2A3
size 5 10 15 20
fold change 10.0 7.5 5.0 2.5 0.0

Figure S4: Transcriptomic analysis of LCA4 patient-derived ROs

(A) Estimation of different cell types and proportions in isogenic control ROs (CON), LCA4 patient-derived untreated ROs (UNT) and LCA4 patient-derived AAV treated ROs (AAV) by cell type deconvolution (MuSiC v.1.0.0) of Raw Bulk RNAseq count data.[27] Publicly available single cell RNAseq data from similar-aged retinal organoids was used as a reference for MuSiC.[28] Statistical significance ($p < 0.05$) for individual cell types across conditions within each batch was determined by Kruskal-Wallis tests. (B, C) Heatmaps of normalised count values scaled by column for genes of interest from isogenic control (CON) and untreated LCA4 patient-derived (UNT) ROs. Specific genes of interest for retinal cell types (amacrine cells, bipolar cells, cone photoreceptors, ganglion cells, horizontal cells, Müller glia and rod photoreceptors) and the phototransduction/visual cascade are shown in (B) and (C) respectively. (D) Bar chart and (E) netplot of over-representation analysis of cellular compartments enriched in the significantly differentially expressed gene-set comparing untreated LCA4 patient-derived ROs to isogenic control ROs.

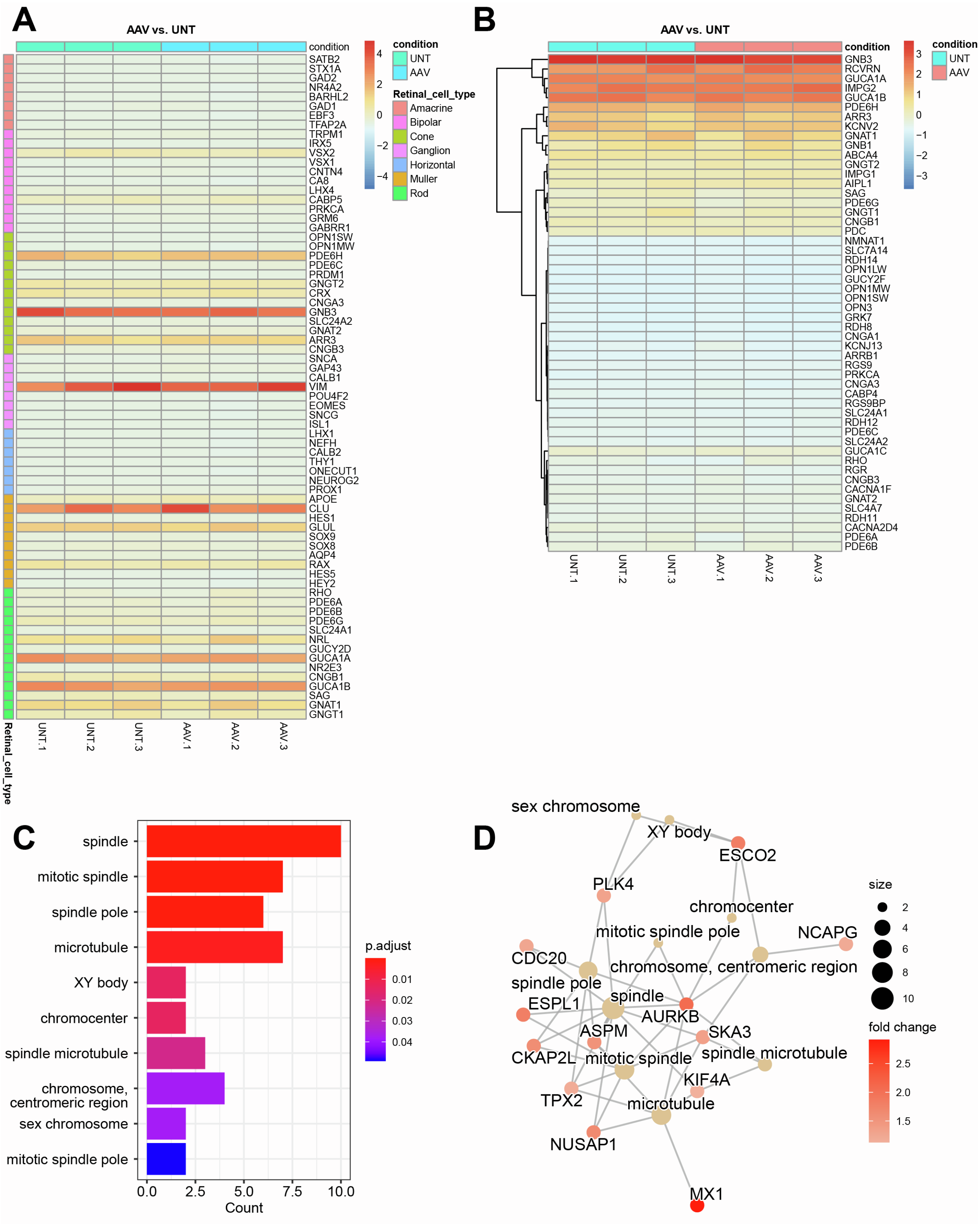
A
AAV vs. UNT
condition
UNT
AAV
Retinal_cell_type
Amacrine
Bipolar
Cone
Ganglion
Horizontal
Muller
Rod
SATB2
STX1A
GAD2
NR4A2
BARHL2
GAD1
EBF3
TFAP2A
TRPM1
IRX5
VSX2
VSX1
CNTN4
CA8
LHX4
CABP5
PRKCA
GRM6
GABRR1
OPN1SW
OPN1MW
PDE6H
PDE6C
PRDM1
GNGT2
CRX
CNGA3
GNB3
SLC24A2
GNAT2
ARR3
CNGB3
SNCA
GAP43
CALB1
VIM
POU4F2
EOMES
SNCG
ISL1
LHX1
NEFH
CALB2
THY1
ONECUT1
NEUROG2
PROX1
APOE
CLU
HES1
GLUL
SOX9
SOX8
AQP4
RAX
HES5
HEY2
RHO
PDE6A
PDE6B
PDE6G
SLC24A1
NRL
GUCY2D
GUCA1A
NR2E3
CNGB1
GUCA1B
SAG
GNAT1
GNGT1
UNT.1
UNT.2
UNT.3
AAV.1
AAV.2
AAV.3
Retinal_cell_type
B
AAV vs. UNT
condition
UNT
AAV
GNB3
RCVRN
GUCA1A
IMPG2
GUCA1B
PDE6H
ARR3
KCNV2
GNAT1
GNB1
ABCA4
GNGT2
IMPG1
AIPL1
SAG
PDE6G
GNGT1
CNGB1
PDC
NMNAT1
SLC7A14
RDH14
OPN1LW
GUCY2F
OPN1MW
OPN1SW
OPN3
GRK7
RDH8
CNGA1
KCNJ13
ARRB1
RGS9
PRKCA
CNGA3
CABP4
RGS9BP
SLC24A1
RDH12
PDE6C
SLC24A2
GUCA1C
RHO
RGR
CNGB3
CACNA1F
GNAT2
SLC4A7
RDH11
CACNA2D4
PDE6A
PDE6B
UNT.1
UNT.2
UNT.3
AAV.1
AAV.2
AAV.3
C
spindle
mitotic spindle
spindle pole
microtubule
XY body
chromocenter
spindle microtubule
chromosome, centromeric region
sex chromosome
mitotic spindle pole
p.adjust
0.01
0.02
0.03
0.04
0.0
2.5
5.0
7.5
10.0
Count
D
sex chromosome
XY body
ESCO2
PLK4
chromocenter
mitotic spindle pole
NCAPG
CDC20
chromosome, centromeric region
spindle pole
spindle
ESPL1
AURKB
ASPM
SKA3
CKAP2L
mitotic spindle
spindle microtubule
KIF4A
TPX2
microtubule
NUSAP1
MX1
size
2
4
6
8
10
fold change
2.5
2.0
1.5

Figure S5: Transcriptomic analysis of LCA4 patient-derived ROs following AAV treatment

(A, B) Heatmaps of normalised count values scaled by column for genes of interest from untreated LCA4 patient-derived (UNT) and treated LCA4 patient-derived ROs (AAV). Specific genes of interest for retinal cell types (amacrine cells, bipolar cells, cone photoreceptors, ganglion cells, horizontal cells, Müller glia and rod photoreceptors) and the phototransduction/visual cascade are shown in (A) and (B) respectively. (C) Bar chart and (D) netplot of over-representation analysis of cellular components enriched in the significantly differentially expressed gene-set comparing AAV treated to untreated LCA4 patient-derived ROs.

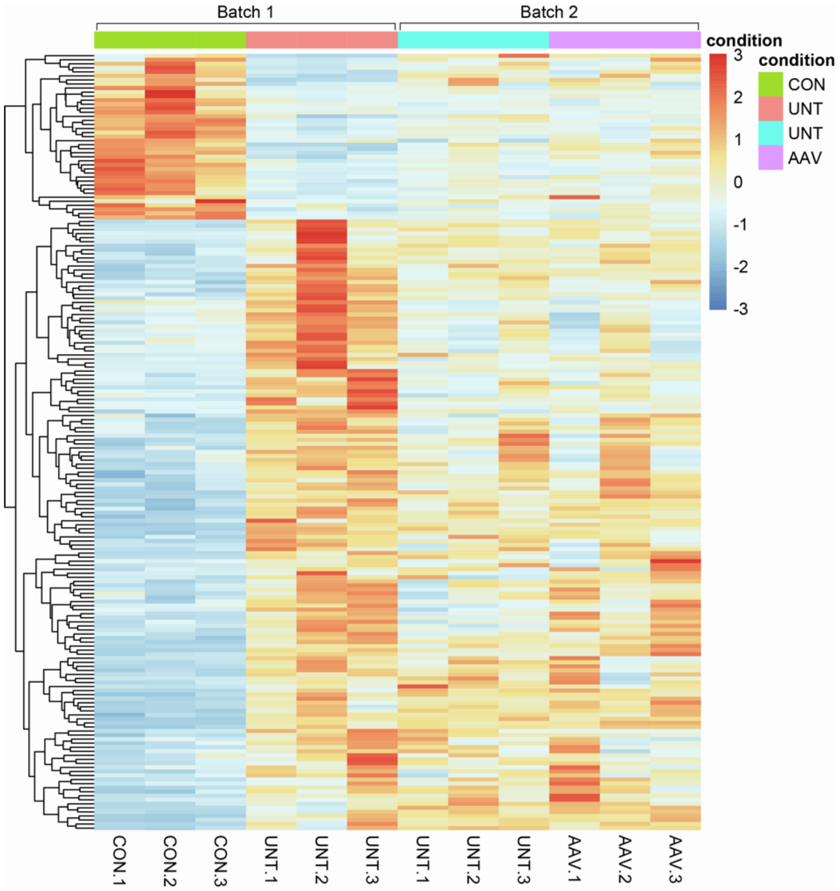

Figure S6: Comparison of gene expression profiles of isogenic control, untreated and AAV treated LCA4 patient ROs.

Heatmap of normalised count values scaled by row for genes of interest from isogenic control (CON), untreated LCA4 patient-derived (UNT) and treated LCA4 patient-derived (AAV) ROs. Genes differentially expressed (padj < 0.05, shrunken LFC > 0.585) between the isogenic control and untreated samples were used to generate the heatmap. Values are represented by red or blue, which indicate relative over- or under-expression of genes, respectively.
