# Supplementary material for: Effective AAV-mediated gene replacement therapy in retinal organoids modeling AIPL1-associated LCA4
Source: Mol Ther Nucleic Acids. 2024 Feb 13;35(1):102148. doi: 10.1016/j.omtn.2024.102148 (PMC10910061; doi:10.1016/j.omtn.2024.102148)
Supplement: Document S2. Article plus supplemental information [file mmc2.pdf]

Molecular Therapy
Nucleic Acids
Original Article

Effective AAV-mediated gene replacement therapy in retinal organoids modeling AIPL1-associated LCA4

Hali Sai,[1,6] Bethany Ollington,[1,6] Farah O. Rezek,[1] Niuzheng Chai,[1] Amelia Lane,[2] Tassos Georgiadis,[2] James Bainbridge,[1,3] Michel Michaelides,[1,3] Almudena Sacristan-Reviriego,[1,4] Pedro R.L. Perdigão,[1,5] Amy Leung,[1] and Jacqueline van der Spuy[1]

[1]University College London Institute of Ophthalmology, University College London, London EC1V 9EL, UK; [2]MeiraGTx, London N1 7NQ, UK; [3]NIHR Moorfields Biomedical Research Centre, London EC1V 2PD, UK; [4]Institute of Clinical Trials and Methodology, University College London, London WC1V 6LJ, UK; [5]Center for Neuroscience and Cell Biology, University of Coimbra, 3004-504 Coimbra, Portugal

Biallelic variations in the *aryl hydrocarbon receptor interacting protein-like 1* (*AIPL1*) gene cause Leber congenital amaurosis subtype 4 (LCA4), an autosomal recessive early-onset severe retinal dystrophy that leads to the rapid degeneration of retinal photoreceptors and the severe impairment of sight within the first few years of life. Currently, there is no treatment or cure for *AIPL1*-associated LCA4. In this study, we investigated the potential of adeno-associated virus-mediated *AIPL1* gene replacement therapy in two previously validated human retinal organoid (RO) models of LCA4. We report here that photoreceptor-specific *AIPL1* gene replacement therapy, currently being tested in a first-in-human application, effectively rescued molecular features of *AIPL1*-associated LCA4 in these models. Notably, the loss of retinal phosphodiesterase 6 was rescued and elevated cyclic guanosine monophosphate (cGMP) levels were reduced following treatment. Transcriptomic analysis of untreated and AAV-transduced ROs revealed transcriptomic changes in response to elevated cGMP levels and viral infection, respectively. Overall, this study supports AIPL1 gene therapy as a promising therapeutic intervention for LCA4.

INTRODUCTION

Leber congenital amaurosis (LCA) is among the most severe and the earliest forms of inherited retinal degeneration (IRD) with an estimated prevalence of 2–3 per 100,000 births.[1] Primarily inherited in an autosomal recessive manner, LCA is characterized by severe sight impairment and abnormal electroretinographic recordings during early childhood.[1] Additional clinical features include attenuation of the retinal blood vessels, optic disc pallor, and nyctalopia.[1] It is genetically heterogeneous and to date, 26 genes have been identified to be linked to LCA.[2] Among these, variations in the *aryl hydrocarbon receptor interacting protein-like 1* (*AIPL1*) gene are estimated to account for 5%–7% of all LCA, resulting in the most severe form of the disease, LCA subtype 4 (LCA4).[3,4]

AIPL1 is a 41-kb gene comprising six exons located on chromosome 17.[5] It encodes a 384 amino acid protein, AIPL1, which is exclusively detected in retinal photoreceptors and the pineal gland.[4,6,7] Functionally, AIPL1 is a photoreceptor-specific co-chaperone that plays an essential role in the assembly and folding of photoreceptor-specific phosphodiesterase 6 (PDE6) together with the molecular chaperone HSP90 in the rod and cone photoreceptors.[8–11] Upon light stimulation, activation of PDE6 hydrolyses cyclic guanosine monophosphate (cGMP) to GMP, which is crucial for light-mediated cell hyperpolarization in the phototransduction cascade.[12,13] Mouse studies have shown that the reduction or absence of Aipl1 protein results in a reduction in Pde6 subunits and elevation of cGMP, which consequently leads to rapid degeneration of rod and cone photoreceptors.[8,10,14,15] There is currently no treatment for this clinical condition and there is therefore an urgent need to develop effective therapies.

Adeno-associated viral (AAV) vector-mediated gene replacement therapies are gaining popularity as they are relatively safe, effective in both proliferating and postmitotic target cells, and can provide prolonged transgene expression.[16,17] AAV-based gene replacement therapy for LCA patients with variations in the *RPE65* gene was granted approval from the U.S. Food and Drug Administration as the first gene therapy for an IRD.[18] AAV-AIPL1 treatment, with transgene expression driven by the cytomegalovirus,[19,20] rhodopsin (Rho),[20] or human Rho kinase (hRK)[21] promoters in combination with the AAV2/2,[19] AAV5,[21] or AAV2/8[19,20] serotypes, has been investigated in LCA4 animal models where it was able to effectively rescue the photoreceptor degeneration.

Recent advances in stem cell-derived 3D retinal organoids (ROs) have provided a model that is well suited for mimicking human

Received 5 September 2023; accepted 12 February 2024;
https://doi.org/10.1016/j.omtn.2024.102148.

[6]These authors contributed equally

Correspondence: Jacqueline van der Spuy, University College London Institute of Ophthalmology, University College London, London EC1V 9EL, UK.
E-mail: j.spuy@ucl.ac.uk

This is an open access article under the CC BY license (http://creativecommons.org/licenses/by/4.0/).

retinal development, with defined layers of inner nuclear layer, outer plexiform layer, and outer nuclear layer (ONL), as well as photoreceptor inner segments (IS) and outer segments (OS).[22,23] ROs have further enabled the investigation of pathological processes of retinal disorders and are a valuable platform for testing the efficacy of potential therapies in human photoreceptor cells. To date, three studies have investigated *AIPL1*-associated LCA4 using human RO models.[24–26] In patient-derived LCA4 models, somatic cells from LCA4 patients, including a patient homozygous for the most common LCA4-associated *AIPL1* variation, c.834G>A, p.W278X, have been reprogrammed to induced pluripotent stem cells (iPSCs) and differentiated to ROs.[24,25] Moreover, CRISPR-Cas9 gene editing has been used to generate an AIPL1 knockout (KO) iPSC line from which ROs have been differentiated.[26] All the iPSC-derived RO models have recapitulated the molecular features evident in the animal models, including a reduction in AIPL1, PDE6α, and PDE6β, accompanied by an elevation in cGMP levels. In addition, it has been reported that the treatment of iPSC-ROs harboring the c.834G>A, p.W278X variation with the translational readthrough inducing drug PTC124 partially restores AIPL1 and PDE6, but not cGMP levels.[25]

To address the need for the development of more efficient therapeutic approaches for LCA4, here we describe AAV-mediated *AIPL1* gene augmentation therapy in LCA4 patient-derived and AIPL1 KO ROs. We found highly efficient restoration of AIPL1, PDE6α, PDE6β, and cGMP levels with AAV treatment. Our study, therefore, provides crucial data in support of future gene therapy approaches for children affected by LCA4.

RESULTS

AAV gene therapy rescued AIPL1 expression in retinal photoreceptors

Two distinct, previously validated LCA4 disease models were used in this study. In the first isogenic LCA4 disease model, CRISPR-Cas9 non-homologous end-joining was used to KO AIPL1 in iPSCs derived from fibroblasts (AIPL1 KO).[26] In the second model, iPSCs were derived from renal epithelial cells from an LCA4 patient homozygous for c.834G>A, p.W278X.[25] The homozygous mutation was corrected by CRIPSR-Cas9 homology directed repair in the patient iPSCs to generate an isogenic control (CON).[25] ROs were differentiated from the AIPL1 KO and LCA4 patient iPSC lines, as previously described.[25,26] LCA4 patient RO and AIPL1 KO ROs were transduced with 1×10^{11} AAV7m8.*hRKp.AIPL1* viral particles at day 196 of differentiation and analyzed 14 days (2 weeks) and 35 days (5 weeks) after transduction at day 210 and day 231 of differentiation, respectively. First, the protein abundance of AIPL1 was investigated with respect to rod and cone photoreceptors (Figure 1). In both LCA4 disease models, endogenous AIPL1 was detected in Rho-positive rod photoreceptors (Figure 1A) and cone arrestin-positive cone photoreceptors (Figure 1B) of the ONL in the isogenic CON ROs at all time points analyzed. No endogenous AIPL1 was detected in the rod (Figure 1A) or cone (Figure 1B) photoreceptors in the untreated (UNT) AIPL1 KO ROs or LCA4 patient ROs. After AAV7m8.*hRKp.AIPL1* treatment (AAV), both models saw increased positive AIPL1 detection in Rho-positive rods (Figure 1A) and cone arrestin-positive cones (Figure 1B). While there seemed to be a low level of AIPL1 recovery in the ONL, AIPL1 expression was prominent in a subpopulation of individual cells. In the AIPL1 KO model, AIPL1 was prominently detected in 10.9% ± 0.5% (day 210) and 12.1% ± 0.5% (day 231) of Rho-positive rod photoreceptors, and in 12.8% ± 0.4% (day 210) and 13.4% ± 0.6% (day 231) of cone arrestin-positive cone photoreceptors. In the LCA4 patient model, AIPL1 was prominently detected in 11.4% ± 0.9% (day 210) and 15.4% ± 0.6% (day 231) of Rho-positive rod photoreceptors, and in 10.8% ± 1.0% (day 210) and 14.3% ± 0.7% (day 231) of cone arrestin-positive cone photoreceptors. AIPL1 was detected throughout both rod and cone photoreceptor cells with the exclusion of the photoreceptor OSs (Figure S1). The results show that expression of the *AIPL1* transgene driven by the hRK promoter (hRKp) led to detectable AIPL1 levels in rod and cone photoreceptors 14 days (2 weeks) after transduction in both AIPL1 KO and patient-derived ROs, which was sustained 35 days (5 weeks) after transduction.

AAV gene therapy rescued rod PDE6 and cGMP levels

We next investigated rod PDE6α (Figure 2A) and rod PDE6β (Figure 2B) in the AIPL1 KO and LCA4 patient models. As previously reported in both LCA4 models,[25,26] PDE6α and PDE6β were localized to the presumptive OS of rod photoreceptors in the isogenic CONs, but were undetectable in UNT AIPL1 KO and LCA4 patient ROs at all time points (Figures 2A and 2B). However, following AAV7m8.*hRKp.AIPL1* transduction (AAV), a remarkable recovery of PDE6α and PDE6β was observed in both models, indicating that AIPL1 function was restored in the ONL (Figures 2A and 2B). The recovery of PDE6α and PDE6β was evident 14 days (2 weeks) after transduction and continued significant rescue was observed 35 days (5 weeks) after transduction (Figures 2A and 2B). Interestingly, there seemed to be some mislocalization of PDE6 protein after AAV treatment, with PDE6 more frequently also observed in the IS and ONL, in addition to the presumptive OS. Pooled whole ROs from the AIPL1 KO model were examined by western blot (Figure S2A). AIPL1 was abundantly detected in isogenic CONs, but absent in AIPL1 KO ROs. PDE6α was also significantly reduced in AIPL1 KO ROs ($p = 0.001$) compared with the isogenic CON. Partial recovery of both AIPL1 and PDE6α protein levels were detected in AAV-transduced AIPL1 KO ROs by western blotting. No difference was observed in the levels of recoverin, used as a CON for photoreceptor cells, confirming that the changes in AIPL1 and PDE6α levels were not related to photoreceptor abundance. As reported previously,[24–26] there was no significant change in the expression of PDE6 transcripts (*PDE6C*, *PDE6H*, *PDE6A*, *PDE6B*, and *PDE6G*) in the AIPL1 KO (Figure S2B) and AIPL1 patient ROs (Figure S2C) by qPCR compared with the respective isogenic CONs, and AAV transduction did not significantly alter these transcript levels compared with the UNT ROs (Figures S2B and S2C), indicating the post-transcriptional rescue of PDE6 following AAV treatment. Finally, cGMP levels were quantified in individual whole ROs to investigate the downstream effects of PDE6 recovery in the AIPL1 KO (Figure 2C) and LCA4 patient

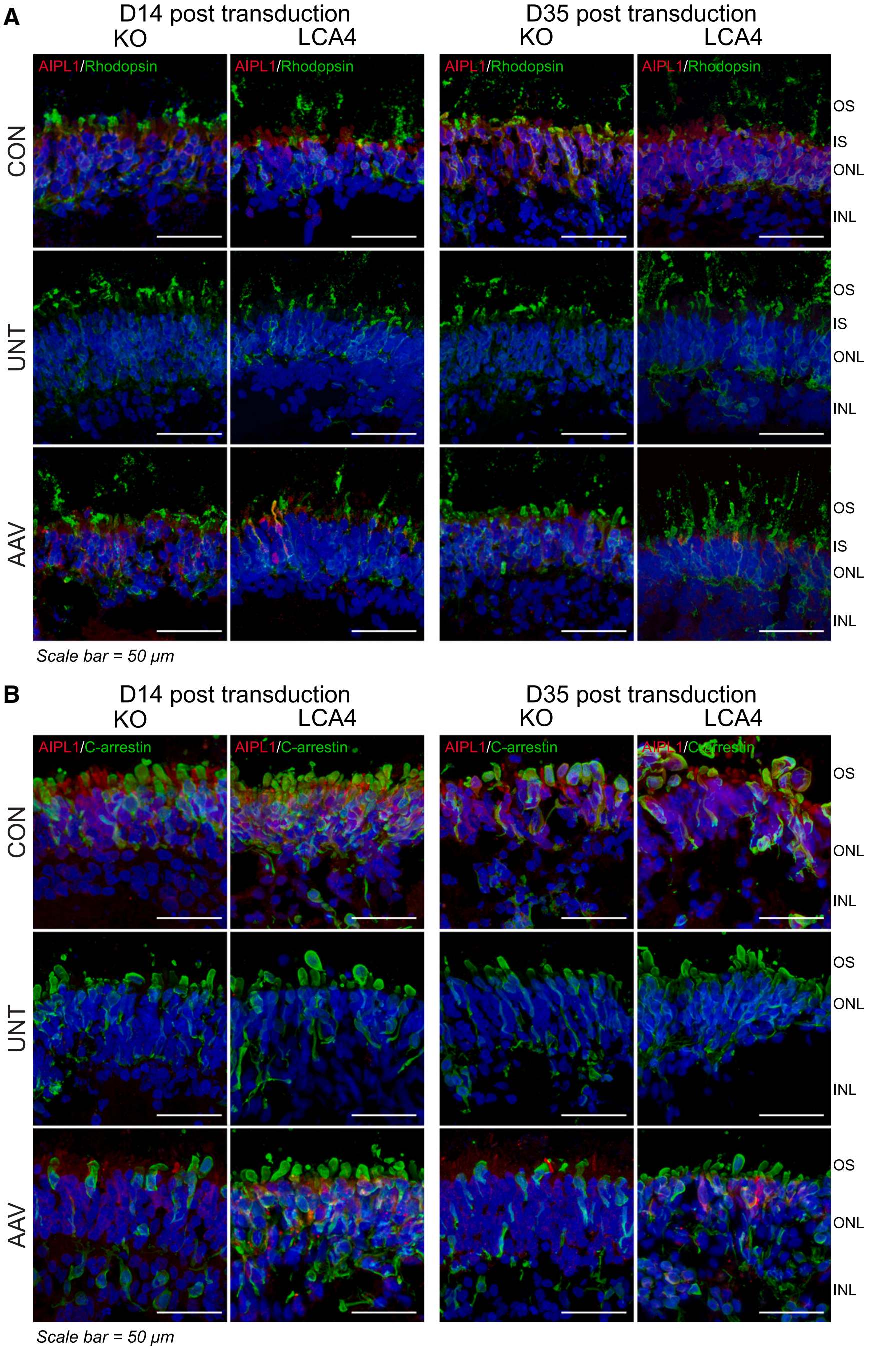

Figure 1. AIPL1 expression in retinal photoreceptors is rescued by AAV gene therapy

(A) Expression of AIPL1 (red) and Rho (green) and (B) AIPL1 (red) and cone arrestin (green) in AIPL1 KO and LCA4 patient-derived (LCA4) ROs transduced with AAV7m8.*hRKp.AIPL1* at day 196 of differentiation and analyzed D14 and D35 after transduction. In both models, AIPL1/Rho and AIPL1/cone arrestin were investigated in the isogenic CON, UNT, and AAV-treated (AAV) ROs. Images are representative of multiple images from at least n = 3 independent ROs for each condition. Nuclei are labeled with diamidino-2-phenylindole (DAPI) (blue). INL, inner nuclear layer; ONL, outer nuclear layer; IS, inner segments; OS, outer segments. Scale bars, 50 μm.

(Figure 2D) models. In both models, the loss of AIPL1 and rod PDE6 subunits produced a significant increase in cGMP levels (p = 0.0285, KO model; p = 0.007, patient model), which was rescued by AAV treatment, indicating functional rescue of the disease phenotype.

The effects of AAV gene therapy persisted for at least 70 days

To understand the duration of the AAV-mediated rescue, LCA4 patient ROs were transduced with AAV7m8.*hRKp.AIPL1* at day 161 of differentiation and analyzed at 14 days (2 weeks), 35 days (5 weeks) (data not shown), and 70 days (10 weeks) after transduction at day 175, day 210, and day 231 of differentiation, respectively (Figure 3). At 14 days (D14) and 70 days (D70) after transduction, AIPL1 was abundant in the ONL of the isogenic CON, but could not be detected in the UNT LCA4 patient ROs (Figure 3A). The transduction of LCA4 patient ROs (AAV) rescued AIPL1 at D14 after transduction and the rescue was sustained 10 weeks (D70) after transduction, with AIPL1 expression detected in a subset of cells at this time point. As shown before, PDE6β was expressed in the presumptive photoreceptor OS in the isogenic CONs, but was absent in LCA4 patient ROs (UNT) (Figure 3B). The rescue of PDE6β was evident at D14 after transduction and was sustained after 10 weeks (D70) (AAV). At day 70 (10 weeks) after transduction, mislocalization of PDE6β to the photoreceptor IS and ONL was noticeable. There was no significant change in the expression of PDE6 transcripts (*PDE6C, PDE6H, PDE6A, PDE6B*, and *PDE6G*), with the exception of a significant increase in *PDE6B* expression D14 post transduction in the UNT and treated LCA4 ROs and of *PDE6G* expression D35 post transduction in the UNT LCA4 ROs, compared with the isogenic CONs (Figure S3). cGMP levels were quantified in individual whole ROs 10 weeks after transduction (Figure 3C). cGMP levels were significantly increased in the LCA4 patient ROs (UNT) compared with the isogenic CONs (p = 0.0056), with AAV treatment (AAV) reducing the cGMP levels to CON levels. In summary, the sustained rescue of AIPL1 function and PDE6 recovery in photoreceptor OSs could be observed 70 days (10 weeks) after transduction of LCA4 patient ROs.

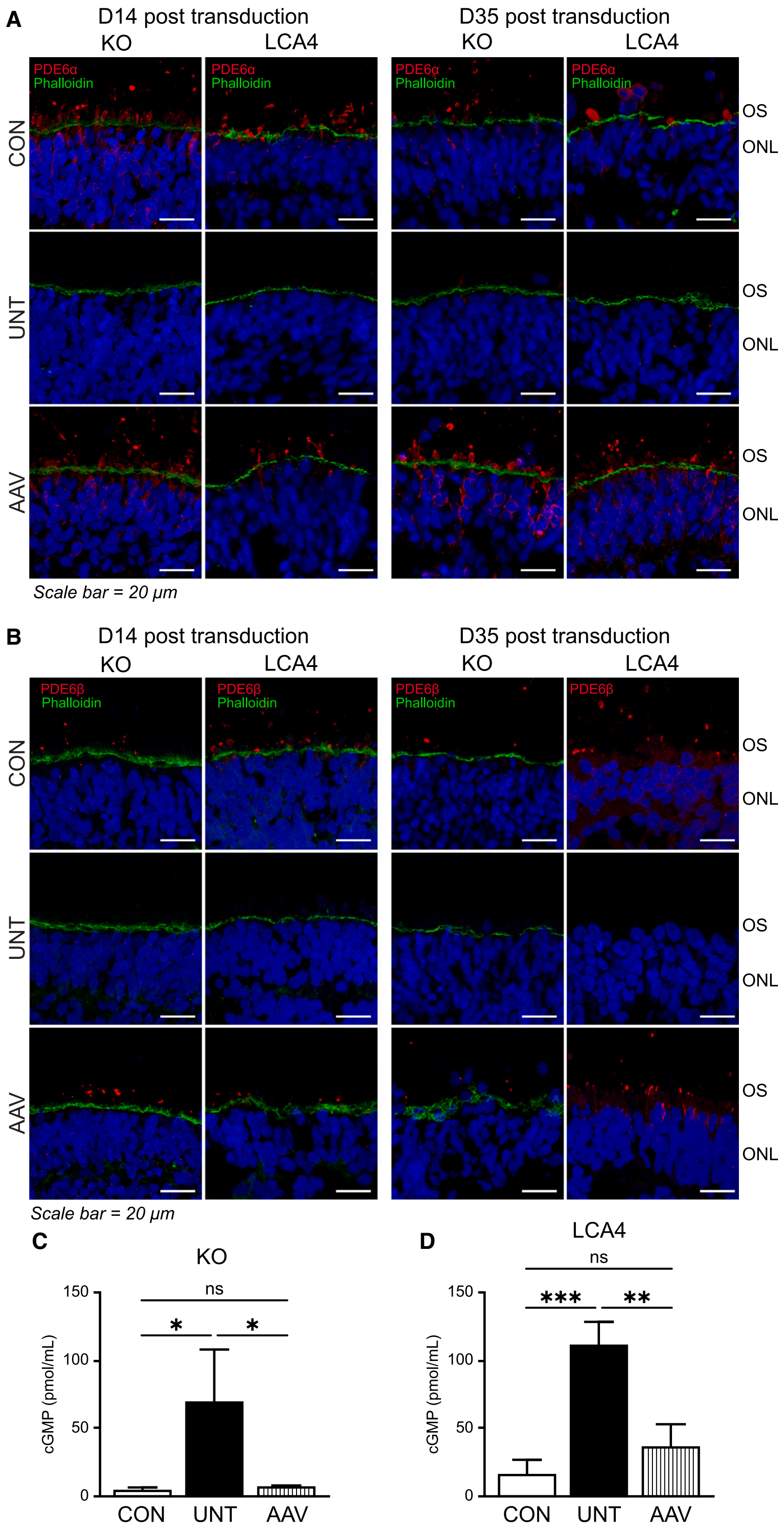

Figure 2. Rod PDE6 and cGMP levels are rescued by AAV gene therapy

(A) Expression of PDE6α (red) and phalloidin (green) and (B) PDE6β (red) and phalloidin (green), as indicated, in isogenic CONs, untreated AIPL1 KO and LCA4 patient (LCA4) ROs (UNT), and AIPL1 KO and LCA4 patient (LCA4) ROs transduced with AAV7m8.*hRKp.AIPL1* (AAV) at day 196 of differentiation and analyzed at D14 and D35 after transduction. Images are representative of multiple images from at least n = 3 independent ROs for each condition. Nuclei are labeled with diamidino-2-phenylindole (DAPI) (blue). ONL, outer nuclear layer; OS, outer segments. Scale bars, 20 μm. (C) cGMP ELISA of individual whole ROs from the AIPL1 KO model and (D) the LCA4 patient (LCA4) model. cGMP levels were measured in the isogenic CON, UNT, and AAV-treated (AAV) ROs 35 days (5 weeks) after transduction at day 231 of differentiation. n = 3 independent ROs per sample, statistical significance determined by One-way ANOVA where *, *,* and *** denotes a p values < 0.05, 0.01, and 0.005 respectively. ns, not significant.

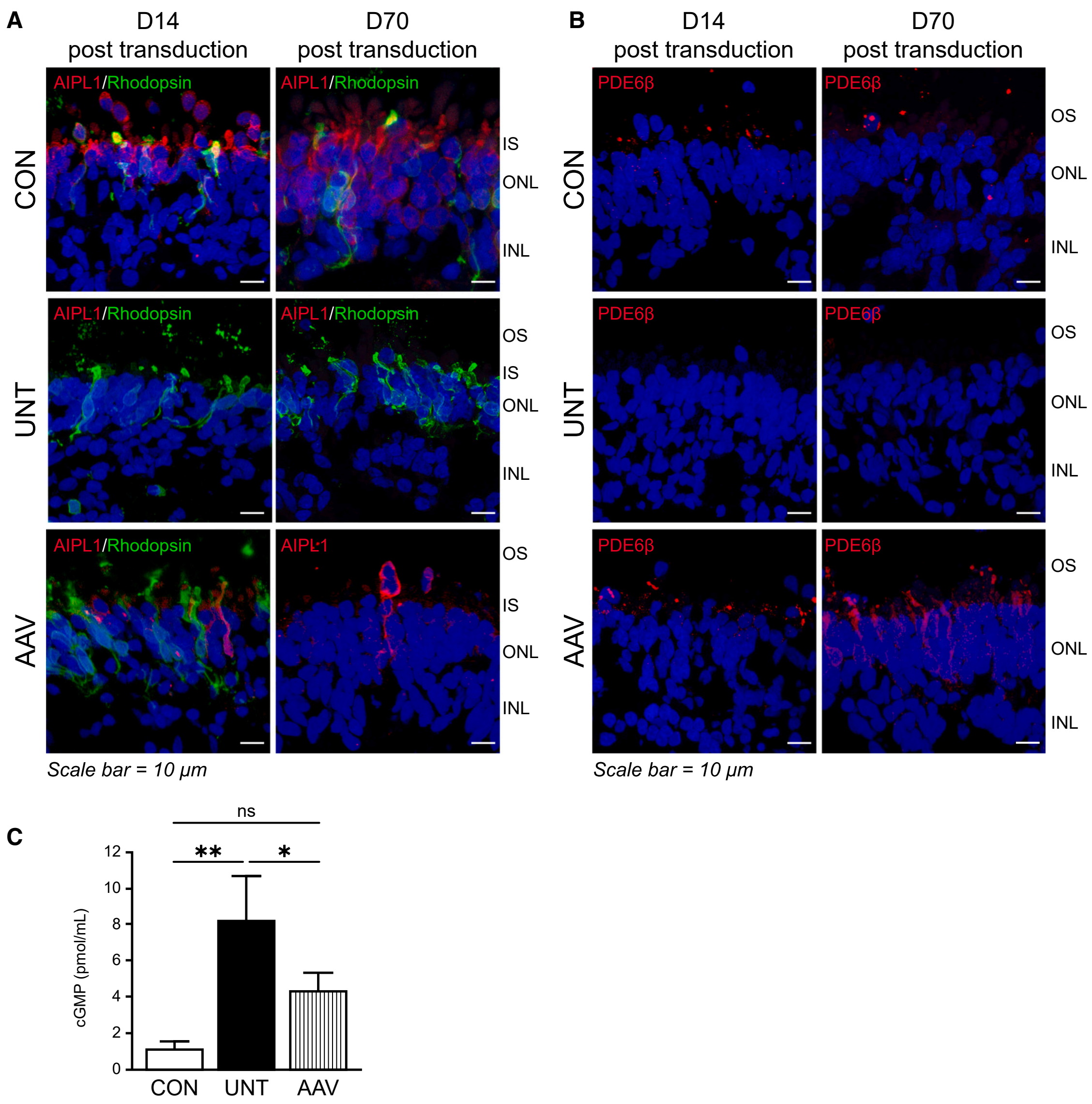

Figure 3. AAV gene therapy leads to sustained phenotypic rescue

(A) Expression of AIPL1 (red) and Rho (green), as indicated, and (B) PDE6β (red) in isogenic CON, UNT LCA4 patient ROs, and LCA4 patient ROs transduced with AAV7m8.*hRKp.AIPL1* (AAV) at day 161 of differentiation and analyzed at D14 and D70 after transduction. Images are representative of multiple images from at least n = 3 independent ROs per condition. Nuclei are labeled with diamidino-2-phenylindole (DAPI) (blue). INL, inner nuclear layer; ONL, outer nuclear layer, OS; outer segments; IS, inner segments. Scale bars, 10 μm. (C) cGMP ELISA of individual whole ROs. cGMP levels were measured in the isogenic CON, UNT, and AAV-treated (AAV) ROs 70 days (10 weeks) after transduction. n = 3 independent ROs per sample, statistical significance determined by One-way ANOVA where * and ** denote a p value < 0.05 and 0.01, respectively. ns, not significant.

Transcriptomic changes in LCA4 patient ROs

We conducted RNA sequencing (RNA-seq) of individual day 231 LCA4 isogenic CON and patient ROs to understand the disease-associated transcriptomic changes (Figure 4). Principal component analysis (PCA) showed distinct and non-overlapping clustering of the LCA4 isogenic CON and UNT LCA4 patient ROs with 45% and 25% of the variance ascribed to PC1 and PC2, respectively (Figure 4A). We estimated the different retinal cell types and proportions in the ROs using single cell RNA-seq data from ROs of a similar age (Figure S4A).[27,28] We found there was no significant difference in the proportions of different retinal cell types ($p > 0.05$; Kruskal-Wallis test) in the LCA4 isogenic CON and UNT patient ROs, except for cone OFF bipolar cells ($p = 0.03$). A heatmap of retinal cell type markers (Figure S4B) and phototransduction markers (Figure S4C) revealed no significant differences between isogenic CON and UNT LCA4 patient ROs (UNT). A volcano plot (adjusted p value (p_{adj}) < 0.05; shrunken

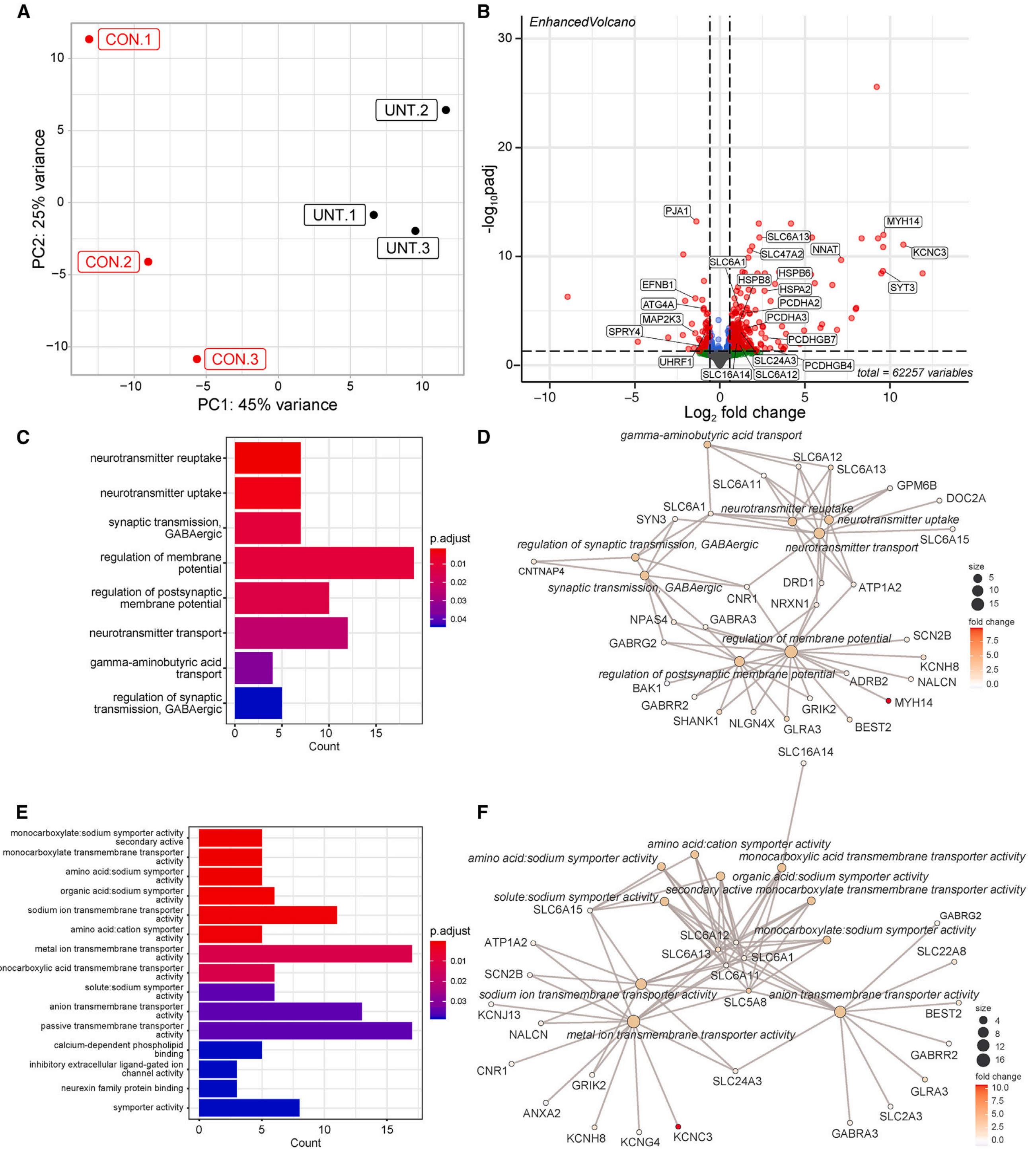

Figure 4. LCA4-associated transcriptional changes affect neuronal signaling and transport

(A) PCA of isogenic CON and LCA4 patient-derived ROs (LCA4) with the percentage of total variance attributed to principal component (PC)1 and PC2 indicated. (B) Volcano plot of DEGs. Significantly upregulated and downregulated genes ($p_{adj} < 0.05$; shrunken LFC > 0.585) are indicated by red dots. (C) Bar chart and (D) netplot of over-representation analysis of biological processes enriched in significantly DEGs. (E) Bar chart and (F) netplot of over-representation analysis of molecular functions enriched in significantly DEGs.

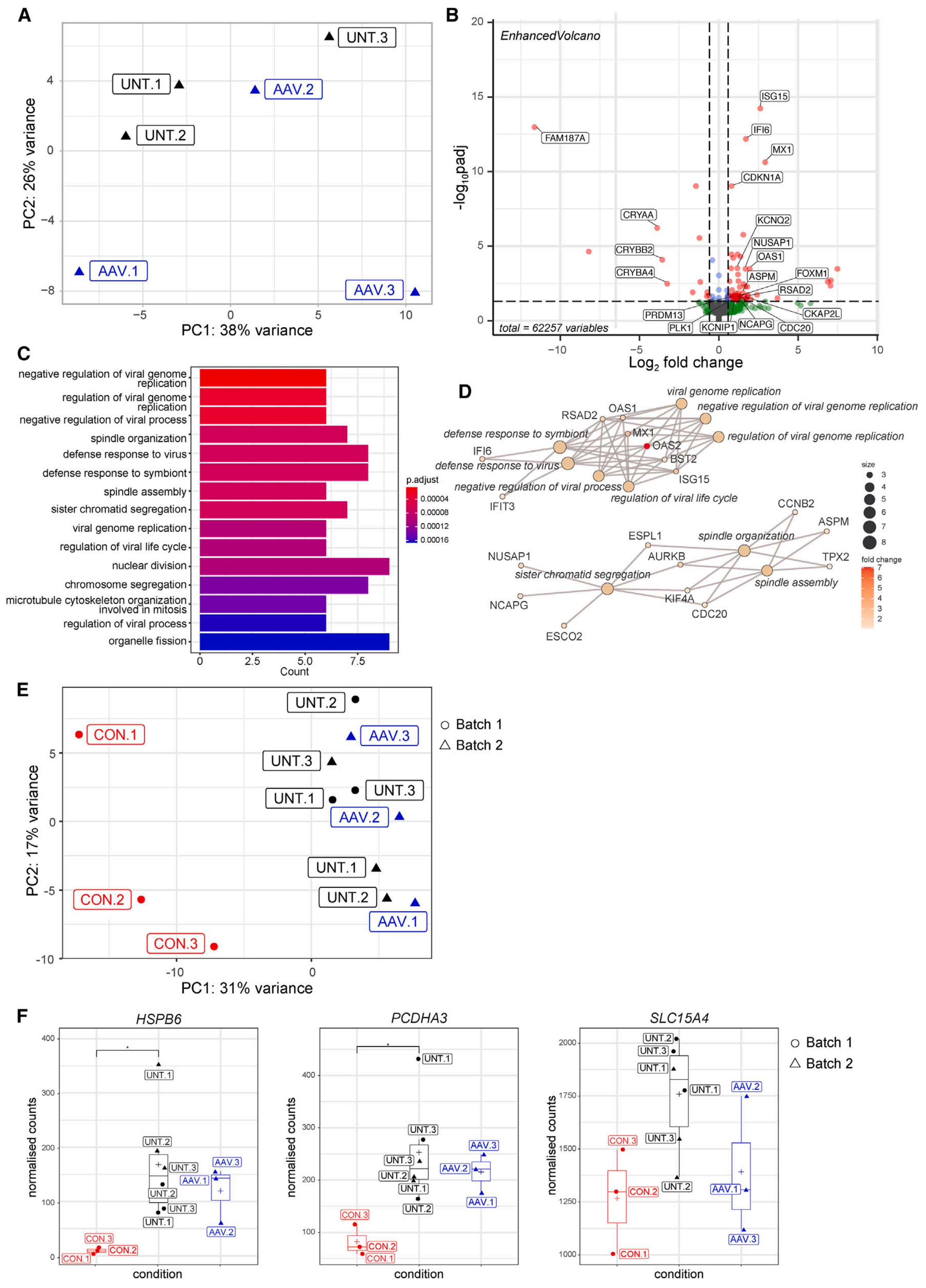

(legend on next page)

log2-fold change (LFC) $\leq$ 0.585) of differentially expressed genes (DEGs) (164 upregulated, 45 downregulated) highlighted the significant upregulation of *Potassium Voltage-Gated Channel Subfamily C Member 3* (*KCNC3*; p_{adj} = 2.5E−11; LFC, 10.8), *Myosin Heavy Chain 14* (*MYH14*; p_{adj} = 1.7E−12; LFC, 9.6), *Synpatotagmin 3* (*SYT3*; p_{adj} = 6.1E−09; LF,C 9.6) and *Neuronatin* (*NNAT*; p_{adj} = 6.2E−10; LFC, 7.1) among the top upregulated genes, in addition to multiple members of the heat shock protein (*HSP*), solute carrier (*SLC*), and protocadherin (*PCDH*) families (Figure 4B) in the LCA4 patient ROs. Gene enrichment analysis of biological processes (Figures 4C and 4D) and cellular compartments (Figures S4D and S4E) revealed dysregulation of pathways related to synaptic regulation and neurotransmission in the LCA4 patient ROs compared with the isogenic CONs, with gene enrichment analysis of molecular function revealing these changes to be primarily mediated by dysregulation of ion channel and transmembrane transporter activity (Figures 4E and 4F).

AAV gene therapy induced an intrinsic viral response

To investigate transcriptomic changes induced by AAV treatment, individual LCA4 UNT and AAV transduced LCA4 ROs were analyzed by RNA-seq (Figure 5). Analysis of the proportion of different retinal cell types revealed there were no significant differences between LCA4 isogenic CONs, LCA4 UNT ROs and AAV treated LCA4 ROs (AAV) ($p > 0.05$; Kruskal-Wallis test), except for cone OFF bipolar cells ($p = 0.03$) (Figure S4A). No significant differences were detected in retinal cell type markers (Figure S5A) or phototransduction markers (Figure S5B) between UNT LCA4 ROs and AAV treated LCA4 ROs (AAV). The number of DEGs was relatively small (51), with only 40 significantly upregulated and 11 significantly downregulated genes. As a consequence, the UNT and AAV-treated ROs did not cluster distinguishably by PCA, although the UNT LCA4 ROs were more similar to one another (Figure 5A). Noticeably, significantly DEGs were related primarily to innate immune or interferon-inducible viral responses, or to the cell cycle (Figure 5B). Among the top significantly upregulated genes related to viral responses, highlighted on a volcano plot, were *MX Dynamin Like GTPase 1* (*MX1*; p_{adj} = 2.1E−10; LFC 2.9), *Interferon-Stimulated Gene 15* (*ISG15*; p_{adj} = 5.9E−14; LFC 2.6), *Interferon Alpha Inducible Protein 6* (*IFI6*; p_{adj} = 6.7E−11; LFC 1.7), and *2′-5′-Oligoadenylate Synthetase 1* (*OAS1*; p_{adj} = 0.0007; LFC 2) (Figure 5B). Similarly, gene enrichment analysis of biological processes highlighted two distinct gene regulatory networks related to the upregulation of viral response pathways and dysregulation of key cell division pathways, respectively, in the AAV-treated ROs compared with the UNT ROs (Figures 5C and 5D), with the latter pathway also highlighted by cell component gene enrichment analysis (Figures S5C and S5D). Finally, we compared the transcriptomic data from the isogenic CON, UNT LAC4 patient and AAV-treated LCA4 patient (AAV) ROs. PCA showed distinct and non-overlapping clustering of the isogenic CONs from both the UNT and treated (AAV) LCA4 patient-derived ROs with 31% and 17% of the variance ascribed to PC1 and PC2, respectively (Figure 5E). Interestingly, the UNT and treated (AAV) LCA4 patient ROs clustered together, indicating that the gene expression profiles of these two groups are similar. This was confirmed by a heatmap of normalized cell counts of genes differentially expressed between the isogenic CON and UNT ROs, showing that the expression profile of DEGs of the treated LCA4 patient-derived ROs (AAV) is most similar to the UNT ROs, particularly within batches (Figure S6). Comparison of the normalized cell counts of individual DEGs similarly highlighted that while there was a general trend toward recovery of gene expression levels in the treated ROs (AAV) toward CON levels, this did not reach significance, as illustrated for *HSPB6*, *PCDH3*, and *SLC15A4* (Figure 5F).

DISCUSSION

In this study, we show effective and sustained rescue of the levels of AIPL1, PDE6, and cGMP in two iPSC-RO models of AIPL1-associated LCA4 after AAV7m8.*hRKp.AIPL1* gene therapy. Our study coincides with a first-in-human application of gene therapy for LCA4, in which affected children have been treated with AAV2/8.*hRKp.AIPL1* via subretinal injection in one eye (M. Michaelides and J. Bainbridge, personal communication, 2023). Our findings of the rescue of AIPL1, PDE6 and cGMP levels as observed in our *in vitro* gene therapy model suggest that *AIPL1* gene replacement therapy may be a valuable approach to treat LCA4 patients.

Notably, as reported previously, there is no overt photoreceptor degeneration in the iPSC-RO models used here, despite the loss of AIPL1 and PDE6 and the elevated levels of cGMP.[25,26] Specifically, we previously reported the normal histological and morphological development of the ROs and normal specification of all retinal cell types in the iPSC-RO models.[25,26] There was, moreover, no change in ONL thickness or TUNEL-positive apoptotic cells in these models. These findings are in agreement with those of a previous study that characterized a patient-derived RO model harboring a homozygous c.256T>C, p.C89R *AIPL1* variation.[24] Lukovic et al. (2020)[24] similarly reported normal retinal cell genesis, differentiation, and cell type specification with preserved retinal morphology, in addition to the lack of reactive gliosis and apoptotic cell death. Interestingly, transcriptomic data from our current study corroborates that of Lukovic et al. (2020),[24] as we observe no

Figure 5. AAV gene therapy induces an innate immune response

(A) PCA of LCA4 patient-derived UNT and AAV-treated (AAV) ROs with the percentage of total variance attributed to principal component (PC)1 and PC2 indicated. (B) Volcano plot of DEGs. Significantly upregulated and downregulated genes ($p_{adj} < 0.05$; shrunken LFC > 0.585) are indicated by red dots. (C) Bar chart and (D) netplot of over-representation analysis of biological processes enriched in significantly DEGs. (E) PCA of isogenic CON, LCA4 patient-derived UNT, and LCA4 patient-derived AAV-treated (AAV) ROs with the percentage of total variance attributed to PC1 and PC2 indicated. (F) Normalized count values for genes of interest in the isogenic CON, UNT LCA4 patient-derived, and treated LCA4 patient-derived (AAV) ROs that were found to be differentially expressed ($p_{adj} < 0.05$; shrunken LFC > 0.585) between the isogenic CON and UNT RO. Differences between each condition (CON, UNT, and AAV) were assessed by One-way ANOVA with a post hoc Tukey honest significant differences test where * denotes a p value < 0.05.

transcriptomic changes in gene expression markers of retinal cell types or phototransduction. We, moreover, do not observe any gene signatures for increased retinal degeneration. The lack of overt photoreceptor degeneration may be due to the fact that ROs faithfully recapitulate *in utero* retinogenesis with the transcriptional profile and retinal cells types of 38 week ROs closely resembling the new-born human retina.[28–32] While ROs mimic the developing human retina, we reported previously using high-resolution optical coherence tomography that the outer retina may be preserved in patients up to 1 year of age.[33] While the exact trigger for retinal degeneration has, therefore, yet to be determined, our transcriptomic data reveal intriguing clues as to the consequences of cGMP elevation preceding the onset of photoreceptor degeneration. Nevertheless, the iPSC-RO models do recapitulate key molecular features of *AIPL1*-associated LCA4, which are rescued by AAV7m8.*hRKp.AIPL1* treatment. Our data suggest that this is a post-transcriptional phenomenon, since no notable changes in PDE6 transcripts or the transcriptome in general were detected either by qPCR or RNA-seq after AAV7m8.*hRKp.AIPL1* treatment.

The increased levels of cGMP observed in our iPSC-RO models are expected to lead to the constitutive opening of cyclic nucleotide-gated channels in the photoreceptor OS, leading to an influx of Ca^{2+} and Na^{+} that, under normal physiological conditions, would be countered by the activity of the $Na^{+}/Ca^{2+}/K^{+}$ exchanger in the OS and the ATP-driven Na^{+}/K^{+} exchanger in the IS.[34] The dysregulation of Ca^{2+} homeostasis and consequences thereof in our LCA4 patient-derived iPSC-ROs is strongly supported by our transcriptomic data that highlighted the dysregulation of pathways related to synaptic transmission and regulation. These data showed the upregulation of genes coding for several members of the solute carrier family of Na^{+}-coupled transporters, including members of the SLC24 family of $Na^{+}/K^{+}/Ca^{2+}$ exchangers. Our data, moreover, showed the upregulation of members of the family of potassium voltage-gated channels, including KCNC members that mediate the voltage-dependent potassium ion permeability of excitable membranes. The genes *SYT3* and *NNAT*, coding for synaptotagmin-3 and neuronatin, were also significantly upregulated in our iPSC-RO model of LCA4, and are involved in Ca^{2+}-dependent exocytosis of secretory vesicles and ion channel regulation during brain development respectively. Interestingly, a decreased expression of bipolar cell postsynaptic proteins (mGluR6, TRPM1) is observed in *Aipl1*$^{-/-}$ mice prior to the onset of photoreceptor degeneration, suggesting abnormal development of bipolar synapses.[35] Cell type deconvolution of our transcriptomic data also suggested an altered proportion of cone OFF bipolar cells in the LCA4 iPSC-RO, warranting further investigation of these signaling pathways. In contrast, our transcriptomic data of the LCA4 patient-derived iPSC-RO treated with AAV7m8.*hRKp.AIPL1* identified the upregulation of genes related primarily to innate immune or interferon-inducible viral responses. In the absence of a humoral or adaptive immune response, the transcriptomic response to viral transduction is likely mediated by the Müller glia cells. While the exact pathways involved in the Müller glia-mediated intrinsic immune response to AAV7m8.*hRKp.AIPL1* transduction require further investigation, the upregulated genes in our transcriptomic data are primarily interferon-stimulated genes. This finding supports the use of steroid prophylaxis in LCA4 patients (M. Michaelides and J. Bainbridge, personal communication, 2023) to mitigate an interferon-mediated response.

We conclude that *AIPL1* gene augmentation therapy rescues key molecular features of the LCA4 phenotype in AIPL1 KO and patient-derived ROs and is a promising treatment approach for children with LCA4.

MATERIALS AND METHODS

Generation of ROs

iPSCs were maintained on Geltrex-coated 6 well plates in mTeSR Plus media (Stemcell, Cambridge, UK) at 37°C in a humidified atmosphere of 5% CO_2 and routinely passaged using Versene (Gibco, Loughborough, UK). Two distinct cell lineages were used in this study, iPSCs derived from renal epithelial cells of an LCA4 patient with a CRISPR-Cas9-corrected isogenic CON[25] and iPSCs derived from commercially available BJ fibroblasts with an isogenic CRISPR-Cas9 *AIPL1* KO.[26] Directed differentiation of iPSCs into 3D ROs was carried out as previously described.[25,26] In brief, differentiation began with iPSCs at 90%–95% confluency, which were cultured in Essential 6 media (Gibco) for 2 days then Neural Induction Media (Advanced DMEM/F12 (1:1), 1% non-essential amino acids, 1% N2 Supplement, 1% GlutaMAX, 1% Antibiotic-Antimycotic (all Gibco)) until development of neuroretinal vesicles (NRVs), typically between day 28 and 42. NRVs were manually excised then cultured further in Retinal Differentiation Media (DMEM/F12 nutrient mix (3:1 ratio; Gibco), 10% fetal bovine serum (Gibco), 2% B27 supplement (without vitamin A; Gibco), 100 μM taurine (Tocris, Abingdon, UK), 2 mM GlutaMAX (Gibco), and 100 U/mL penicillin/streptomycin (Gibco)), with media changes every 2 days. At day 70, cultures were additionally supplemented with 1 μM retinoic acid. At day 84, the cultures were further supplemented with 1% N2 and RA lowered to 0.5 μM. Finally, at day 100, B27 and RA were removed from the medium for the remaining culture period.

Generation of AAV and treatment of ROs

The pAAV-hRK AIPL1 plasmid construction is described in Tan et al. (2009).[19] Recombinant AAV7m8 virus was generated by helper virus-free, triple transfection. HEK293 suspension cells were transfected with pAAV-hRK AIPL1, pRep2/Cap7m8, and pHelper at a ratio of 1:1:2. Seventy-two hours after transfection, AAV particles were purified from the clarified lysate by AAVX affinity chromatography. AAV-containing fractions were concentrated and formulated using a 100K molecular weight cut-off protein concentrator using a Dulbecco's PBS buffer supplemented with Pluronic F68 (0.001%). Genomic titers (vg/mL) were determined by real-time qPCR. LCA4 patient and AIPL1 KO ROs were transduced with 1×10^{11} AAV7m8.*hRKp.AIPL1* virus particles at day 196. The treated ROs were collected 14 days after transduction (day 210 of differentiation) and 35 days after transduction (day 231 of differentiation) for analysis compared with the respective

UNT ROs and isogenic CONs. For the 10 week analysis, LCA4 patient ROs were transduced with 1×10^{11} AAV7m8.*hRKp.AIPL1* virus particles at day 161 and ROs collected 14 days later (day 175 of differentiation) or 70 days later (day 231 of differentiation).

Immunofluorescence and imaging

For fixation, ROs were incubated in 4% paraformaldehyde, 5% sucrose in PBS for 30 min at 4°C, then dehydrated for 1 h each in 6.25%, 12.5%, and 25% sucrose:PBS at 4°C, and finally embedded in Tissue-Tek OCT compound and stored at −80°C. We mounted 7-μm cryosections on SuperFrost Plus slides (Thermo Fisher Scientific, Paisley, UK). Slides were stained for immunocytochemistry by first incubating in blocking solution (10% donkey serum [Sigma Aldrich, Gillingham, UK], 0.01% Triton X [Sigma Aldrich] in PBS) for 1 h at room temperature (RT). Next, slides were incubated with a primary antibody for 1 h (RT; antibody-specific dilutions) (Table S1), washed with PBS, and then incubated with species-specific secondary antibody for 45 min (1:1,000) (Table S1). Slides were then incubated with Alexa Fluor 488 phalloidin (Thermo Fisher Scientific) where indicated for 20 min, and finally stained with 4,6-diamidino-2-phenylindole (DAPI) (2 mg/mL) (Invitrogen, Loughborough, UK) in PBS for 5 min. For mounting, slides were washed three times with PBS, dried at RT, and mounted in Fluorescence Mounting Media (Dako, Agilent, CA, USA). All images were acquired using a Leica Stellaris 5 or Zeiss LSM700 laser-scanning confocal microscope. Images were prepared using Adobe Photoshop, ImageJ, and Adobe Illustrator CS6.

RNA extraction and qPCR

RNA from ROs was extracted using the PicoPure RNA Isolation Kit (Thermo Fisher Scientific) and cDNA synthesis was performed using the High-Capacity cDNA Reverse Transcription Kit (Thermo Fisher Scientific), both as per manufacturer's instructions. Real-time PCR reactions were set up with 2× LabTaq Green Hi Rox Master Mix (Labtech, East Sussex, UK) and validated primers (0.25 pM/μL) (primer sequences in Table S2) and run on an Applied Biosystems (Foster City, CA, USA) QuantStudio 6 Flex real-time PCR system. Gene expression levels were normalized to *CRX* as a consistently expressed photoreceptor-specific reference gene to negate differences in size and cellular make-up between samples. Three biological replicates per sample were used to calculate averages and standard deviation, and statistical significance determined by one-way ANOVA with Tukey's HSD post hoc analysis where *, **, and *** denote a p value of $\leq$ 0.05, $\leq$0.01, and $\leq$0.005, respectively.

Western blotting

Protein was isolated from three to six pooled ROs by incubating in RIPA buffer for 15 min on ice, briefly sonicating, then centrifuging at 4°C for 10 min at 13k rpm. Protein concentration was quantified by Pierce BCA assay (Thermo Fisher Scientific) as per manufacturer's instructions. Protein concentrations were normalized, then mixed 1:1 with 2× SDS PAGE buffer and heated at 95°C for 5 min before running on a 4%–20% gradient gel (Bio-Rad, Watford, UK) at 120 V for 2 h. Protein was transferred to a nitrocellulose membrane using standard protocols, and successful transfer confirmed by Ponceau stain. Membranes were first blocked in 5% skim milk powder in PBS-T for 1 h at RT, then incubated overnight in primary antibody (Table S1) at 4°C. Next, samples were incubated in species-specific HRP-conjugated secondary antibody for 1 h, then developed using Clarity MAX Western ECL (Bio-Rad) for 5 min. Membranes were imaged using a ChemiDoc MP Imaging System (Bio-Rad). Images were formatting using Image Lab (Bio-Rad), which was also used to calculated band density. Statistical significance was determined by one-way ANOVA with Tukey's HSD post hoc analysis where *, **, and *** denote a p value $\leq$ 0.05, $\leq$0.01, and $\leq$0.005, respectively.

Quantification of cGMP

cGMP concentrations were calculated for individual whole ROs by ELISA (Cyclic GMP ELISA kit, Cayman Chemicals, Ann Arbor, MI, USA) according to the manufacturer's instructions. In brief, ROs were washed with PBS then incubated in 100 μL 0.1M HCl for 20 min at RT and mechanically homogenized. The samples were centrifuged at 1,000×*g* for 10 min then the supernatant combined with 200 μL ELISA buffer. We used 50 μL of this sample per well. Absorbance was measured at a wavelength of 420 nm. Values given are normalized to volume to give cGMP concentration per whole RO with at least three RO analysed per condition, and statistical significance calculated by one-way ANOVA with Tukey's HSD post hoc analysis where *, **, and *** denote a p value $\leq$ 0.05, $\leq$0.01, and $\leq$ 0.005, respectively.

RNA-seq

Total RNA was extracted from individual day 230 (week 33) ROs (three ROs per condition in each batch) using PicoPure RNA Isolation Kit (Applied Biosystems) and the RNA concentration determined by NanoDrop spectrophotometer (Thermo Fisher Scientific) quantification. RNA-seq was carried out at a read depth of 20-30million reads per sample by GeneWiz (Azenta Life Sciences); 600 ng total RNA per sample was supplied.

RNA-seq analysis

Fastq files containing bulk RNA-seq reads were aligned to the indexed GENCODE v42 hg38 reference transcriptome and genome using Salmon v1.9.0. A list of decoys was created using the GENCODE v42 human primary assemble genome file. This list was then used to generate the Salmon index along with the reference transcriptomes and genomes. Additional options '–validateMappings –gcBias' were passed in for the Salmon alignment.[36,37] All differential gene expression analyses and subsequent gene enrichment analyses were carried out using R v4.2.1 and its associated packages.[38] Quantification data from Salmon was directly imported using tximeta v1.16.1 into DESeq2 v1.38.3 for differential gene expression analysis.[39,40] The DESeq2 normalisation method was used to normalize the data. The data underwent variance stabilizing transformation before being subject to the PCA. A generalized linear model was fitted to the normalized counts using the negative binominal distribution. p_{adj} were calculated using the Independent Hypothesis Weighting v1.26.0 package to

test the null hypothesis that differential gene expression is less than 1-fold.[41] A p_{adj} of less than 0.05 was used to filter for significant DEGs. For comparing UNT to isogenic CON cell lines, the isogenic samples were used as the reference level. UNT samples were used as the reference level when AAV-treated samples were compared with UNT samples. Shrunken LFC were estimated using apeglm v1.20.0.[42] These shrunken LFC values were used for producing the volcano plot with EnhancedVolcano v1.16.0 ($p_{adj} < 0.05$; shrunken LFC > 0.585), and for ranking the DEGs based on shrunken LFC.[43] The RStudio package Pheatmap v1.0.12 was used to generate heatmaps, where normalized count values scaled by column were plotted for genes of interest. Gene enrichment analysis was carried out using clusterProfiler v4.6.0,[44,45] where a p_{adj} of less than 0.05 and shrunken LFC values of 0.585 and 1 were considered significant for the UNT versus isogenic CON and the UNT versus treated comparisons, respectively. Raw bulk RNA-seq count data from Salmon was also used for cell type deconvolution using MuSiC v1.0.0 to estimate the different cell types and proportions in the ROs.[27] Single cell RNA-seq data from ROs of similar age to our samples was obtained from Cowan et al. (2020) to be used as a reference.[28] Kruskal-Wallis tests were also carried out to check if individual cell types varied significantly ($p < 0.05$) across conditions within each batch.

DATA AND CODE AVAILABILITY

The RNA-seq data generated in this study will be uploaded to the National Center for Biotechnology Information (NCBI) Gene Expression Omnibus (GEO) or Sequence Read Archive (SRA).

SUPPLEMENTAL INFORMATION

Supplemental information can be found online at https://doi.org/10.1016/j.omtn.2024.102148.

ACKNOWLEDGMENTS

The authors gratefully acknowledge funding from the Medical Research Council (MR/P02582X/1) (to J.v.d.S.) and the Rosetrees Trust (A2596) (to J.v.d.S.).

AUTHOR CONTRIBUTIONS

Conceptualization, J.v.d.S.; Data curation, N.C. and F.O.R.; formal analysis, H.S., B.O., N.C., F.O.R., and J.v.d.S.; funding acquisition, J.v.d.S.; investigation, H.S., B.O., N.C., F.O.R, P.R.L.P., A.S-R., and A. Leung; methodology, H.S., B.O., P.R.L.P., A.S-R., and A.Leung; project administration, J.v.d.S.; resources, A.Lane, T.G., J.B., and M.M.; supervision, J.v.d.S; software, N.C. and F.O.R.; validation, H.S., B.O., P.R.L.P., and A.S.-R.; visualization, H.S.; B.O., N.C., F.O.R., and J.v.d.S.; writing – original draft, B.O., H.S., N.C., F.O.R., A.Lane, and J.v.d.S.; writing – review and editing, H.S., B.O., F.O.R., N.C., A.Lane, T.G., P.R.L.P, A.S-R., J.B., M.M., A.Leung.

DECLARATION OF INTERESTS

A.Lane and T.G. are employees and stockholders of MeiraGTx. M.M. is a consultant and has equity in MeiraGTx.

REFERENCES

1. den Hollander, A.I., Roepman, R., Koenekoop, R.K., and Cremers, F.P.M. (2008). Leber congenital amaurosis: genes, proteins and disease mechanisms. Prog. Retin. Eye Res. *27*, 391–419.
2. RetNet. Retinal Information Network. https://web.sph.uth.edu/RetNet/.
3. Dharmaraj, S., Leroy, B.P., Sohocki, M.M., Koenekoop, R.K., Perrault, I., Anwar, K., Khaliq, S., Devi, R.S., Birch, D.G., De Pool, E., et al. (2004). The Phenotype of Leber Congenital Amaurosis in Patients With AIPL1 Mutations. Arch. Ophthalmol. *122*, 1029–1037.
4. Sohocki, M.M., Bowne, S.J., Sullivan, L.S., Blackshaw, S., Cepko, C.L., Payne, A.M., Bhattacharya, S.S., Khaliq, S., Qasim Mehdi, S., Birch, D.G., et al. (2000). Mutations in a new photoreceptor-pineal gene on 17p cause Leber congenital amaurosis. Nat. Genet. *24*, 79–83.
5. Chr17: 6,423K,436K - Genome Data Viewer - NCBI. https://www.ncbi.nlm.nih.gov/genome/gdv/browser/genome/?id=GCF_000001405.4.
6. van der Spuy, J., Chapple, J.P., Clark, B.J., Luthert, P.J., Sethi, C.S., and Cheetham, M.E. (2002). The Leber congenital amaurosis gene product AIPL1 is localized exclusively in rod photoreceptors of the adult human retina. Hum. Mol. Genet. *11*, 823–831.
7. van der Spuy, J., Kim, J.H., Yu, Y.S., Szel, A., Luthert, P.J., Clark, B.J., and Cheetham, M.E. (2003). The expression of the Leber congenital amaurosis protein AIPL1 coincides with rod and cone photoreceptor development. Invest. Ophthalmol. Vis. Sci. *44*, 5396–5403.
8. Liu, X., Bulgakov, O.V., Wen, X.H., Woodruff, M.L., Pawlyk, B., Yang, J., Fain, G.L., Sandberg, M.A., Makino, C.L., and Li, T. (2004). AIPL1, the protein that is defective in Leber congenital amaurosis, is essential for the biosynthesis of retinal rod cGMP phosphodiesterase. Proc. Natl. Acad. Sci. USA *101*, 13903–13908.
9. Hidalgo-de-Quintana, J., Evans, R.J., Cheetham, M.E., and van der Spuy, J. (2008). The Leber congenital amaurosis protein AIPL1 functions as part of a chaperone heterocomplex. Invest. Ophthalmol. Vis. Sci. *49*, 2878–2887.
10. Kolandaivelu, S., Huang, J., Hurley, J.B., and Ramamurthy, V. (2009). AIPL1, a Protein Associated with Childhood Blindness, Interacts with α-Subunit of Rod Phosphodiesterase (PDE6) and Is Essential for Its Proper Assembly. J. Biol. Chem. *284*, 30853–30861.
11. Sacristan-Reviriego, A., Bellingham, J., Prodromou, C., Boehm, A.N., Aichem, A., Kumaran, N., Bainbridge, J., Michaelides, M., and van der Spuy, J. (2017). The integrity and organization of the human AIPL1 functional domains is critical for its role as a HSP90-dependent co-chaperone for rod PDE6. Hum. Mol. Genet. *26*, 4465–4480.
12. Cote, R.H. (2021). Photoreceptor phosphodiesterase (PDE6): activation and inactivation mechanisms during visual transduction in rods and cones. Pflügers Archiv *473*, 1377–1391.
13. Qureshi, B.M., Behrmann, E., Schöneberg, J., Loerke, J., Bürger, J., Mielke, T., Giesebrecht, J., Noé, F., Lamb, T.D., Hofmann, K.P., et al. (2018). It takes two transducins to activate the cGMP-phosphodiesterase 6 in retinal rods. Open Biol. *8*, 180075.
14. Ramamurthy, V., Niemi, G.A., Reh, T.A., and Hurley, J.B. (2004). Leber congenital amaurosis linked to AIPL1: a mouse model reveals destabilization of cGMP phosphodiesterase. Proc. Natl. Acad. Sci. USA *101*, 13897–13902.
15. Kirschman, L.T., Kolandaivelu, S., Frederick, J.M., Dang, L., Goldberg, A.F.X., Baehr, W., and Ramamurthy, V. (2010). The Leber congenital amaurosis protein, AIPL1, is needed for the viability and functioning of cone photoreceptor cells. Hum. Mol. Genet. *19*, 1076–1087.
16. Bordet, T., and Behar-Cohen, F. (2019). Ocular gene therapies in clinical practice: viral vectors and nonviral alternatives. Drug Discov. Today *24*, 1685–1693.
17. Vandenberghe, L.H., and Auricchio, A. (2012). Novel adeno-associated viral vectors for retinal gene therapy. Gene Ther. *19*, 162–168.
18. Gao, J., Hussain, R.M., and Weng, C.Y. (2020). Voretigene Neparvovec in Retinal Diseases: A Review of the Current Clinical Evidence. Clin. Ophthalmol. *14*, 3855–3869.
19. Tan, M.H., Smith, A.J., Pawlyk, B., Xu, X., Liu, X., Bainbridge, J.B., Basche, M., McIntosh, J., Tran, H.V., Nathwani, A., et al. (2009). Gene therapy for retinitis pigmentosa and Leber congenital amaurosis caused by defects in AIPL1: effective rescue

of mouse models of partial and complete Aipl1 deficiency using AAV2/2 and AAV2/8 vectors. Hum. Mol. Genet. *18*, 2099–2114.

20. Testa, F., Surace, E.M., Rossi, S., Marrocco, E., Gargiulo, A., Di Iorio, V., Ziviello, C., Nesti, A., Fecarotta, S., Bacci, M.L., et al. (2011). Evaluation of Italian Patients with Leber Congenital Amaurosis due to AIPL1 Mutations Highlights the Potential Applicability of Gene Therapy. Invest. Ophthalmol. Vis. Sci. *52*, 5618–5624.

21. Sun, X., Pawlyk, B., Xu, X., Liu, X., Bulgakov, O.V., Adamian, M., Sandberg, M.A., Khani, S.C., Tan, M.-H., Smith, A.J., et al. (2010). Gene Therapy with a Promoter Targeting Both Rods and Cones Rescues Retinal Degeneration Caused by AIPL1 Mutations. Gene Ther. *17*, 117–131.

22. Zhong, X., Gutierrez, C., Xue, T., Hampton, C., Vergara, M.N., Cao, L.H., Peters, A., Park, T.S., Zambidis, E.T., Meyer, J.S., et al. (2014). Generation of three-dimensional retinal tissue with functional photoreceptors from human iPSCs. Nat. Commun. *5*, 4047.

23. Gonzalez-Cordero, A., Kruczek, K., Naeem, A., Fernando, M., Kloc, M., Ribeiro, J., Goh, D., Duran, Y., Blackford, S.J.I., Abelleira-Hervas, L., et al. (2017). Recapitulation of Human Retinal Development from Human Pluripotent Stem Cells Generates Transplantable Populations of Cone Photoreceptors. Stem Cell Rep. *9*, 820–837.

24. Lukovic, D., Artero Castro, A., Kaya, K.D., Munezero, D., Gieser, L., Davó-Martínez, C., Corton, M., Cuenca, N., Swaroop, A., Ramamurthy, V., et al. (2020). Retinal Organoids derived from hiPSCs of an AIPL1-LCA Patient Maintain Cytoarchitecture despite Reduced levels of Mutant AIPL1. Sci. Rep. *10*, 5426.

25. Leung, A., Sacristan-Reviriego, A., Perdigão, P.R.L., Sai, H., Georgiou, M., Kalitzeos, A., Carr, A.J.F., Coffey, P.J., Michaelides, M., Bainbridge, J., et al. (2022). Investigation of PTC124-mediated translational readthrough in a retinal organoid model of AIPL1-associated Leber congenital amaurosis. Stem Cell Rep. *17*, 2187–2202.

26. Perdigão, P.R.L., Ollington, B., Sai, H., Leung, A., Sacristan-Reviriego, A., and van der Spuy, J. (2023). Retinal Organoids from an AIPL1 CRISPR/Cas9 Knockout Cell Line Successfully Recapitulate the Molecular Features of LCA4 Disease. Int. J. Mol. Sci. *24*, 5912.

27. Wang, X., Park, J., Susztak, K., Zhang, N.R., and Li, M. (2019). Bulk tissue cell type deconvolution with multi-subject single-cell expression reference. Nat. Commun. *10*, 380.

28. Cowan, C.S., Renner, M., De Gennaro, M., Gross-Scherf, B., Goldblum, D., Hou, Y., Munz, M., Rodrigues, T.M., Krol, J., Szikra, T., et al. (2020). Cell Types of the Human Retina and Its Organoids at Single-Cell Resolution. Cell *182*, 1623–1640.e34.

29. Collin, J., Queen, R., Zerti, D., Dorgau, B., Hussain, R., Coxhead, J., Cockell, S., and Lako, M. (2019). Deconstructing Retinal Organoids: Single Cell RNA-Seq Reveals the Cellular Components of Human Pluripotent Stem Cell-Derived Retina. Stem Cell. *37*, 593–598.

30. Bell, C.M., Zack, D.J., and Berlinicke, C.A. (2020). Human Organoids for the Study of Retinal Development and Disease. Annu. Rev. Vis. Sci. *6*, 91–114.

31. Sridhar, A., Hoshino, A., Finkbeiner, C.R., Chitsazan, A., Dai, L., Haugan, A.K., Eschenbacher, K.M., Jackson, D.L., Trapnell, C., Bermingham-McDonogh, O., et al. (2020). Single-Cell Transcriptomic Comparison of Human Fetal Retina, hPSC-Derived Retinal Organoids, and Long-Term Retinal Cultures. Cell Rep. *30*, 1644–1659.e4.

32. Dorgau, B., Collin, J., Rozanska, R., Boczonadi, V., Moya-Molina, M., Hussain, R., Coxhead, J., Dhanaseelan, T., Armstrong, L., Queen, R., and Lako, M. (2023). Deciphering the spatio-temporal transcriptional and chromatin accessibility of human retinal organoid development at the single cell level. Preprint at bioRxiv. https://doi.org/10.1101/2023.07.19.549507.

33. Aboshiha, J., Dubis, A.M., van der Spuy, J., Nishiguchi, K.M., Cheeseman, E.W., Ayuso, C., Ehrenberg, M., Simonelli, F., Bainbridge, J.W., and Michaelides, M. (2015). Preserved outer retina in AIPL1 Leber's congenital amaurosis: implications for gene therapy. Ophthalmology *122*, 862–864.

34. Lamb, T.D. (2022). Photoreceptor physiology and evolution: cellular and molecular basis of rod and cone phototransduction. J. Physiol. *600*, 4585–4601.

35. Singh, R.K., Kolandaivelu, S., and Ramamurthy, V. (2014). Early alteration of retinal neurons in Aipl1-/- animals. Invest. Ophthalmol. Vis. Sci. *55*, 3081–3092.

36. Patro, R., Duggal, G., Love, M.I., Irizarry, R.A., and Kingsford, C. (2017). Salmon provides fast and bias-aware quantification of transcript expression. Nat. Methods *14*, 417–419.

37. Frankish, A., Diekhans, M., Ferreira, A.M., Johnson, R., Jungreis, I., Loveland, J., Mudge, J.M., Sisu, C., Wright, J., Armstrong, J., et al. (2019). GENCODE reference annotation for the human and mouse genomes. Nucleic Acids Res. *47*, D766–D773.

38. R Core Team (2022). R: A Language and Environment for Statistical Computing (R Foundation for Statistical Computing). https://www.R-project.org/.

39. Love, M.I., Huber, W., and Anders, S. (2014). Moderated estimation of fold change and dispersion for RNA-seq data with DESeq2. Genome Biol. *15*, 550.

40. Love, M.I., Soneson, C., Hickey, P.F., Johnson, L.K., Pierce, N.T., Shepherd, L., Morgan, M., and Patro, R. (2020). Tximeta: Reference sequence checksums for provenance identification in RNA-seq. PLoS Comput. Biol. *16*, e1007664.

41. Ignatiadis, N., Klaus, B., Zaugg, J.B., and Huber, W. (2016). Data-driven hypothesis weighting increases detection power in genome-scale multiple testing. Nat. Methods *13*, 577–580.

42. Zhu, A., Ibrahim, J.G., and Love, M.I. (2019). Heavy-tailed prior distributions for sequence count data: removing the noise and preserving large differences. Bioinformatics *35*, 2084–2092.

43. Blighe, K. (2018). EnhancedVolcano. Bioconductor. https://doi.org/10.18129/B9.BIOC.ENHANCEDVOLCANO.

44. Yu, G., Wang, L.G., Han, Y., and He, Q.Y. (2012). clusterProfiler: an R package for comparing biological themes among gene clusters. OMICS *16*, 284–287.

45. Wu, T., Hu, E., Xu, S., Chen, M., Guo, P., Dai, Z., Feng, T., Zhou, L., Tang, W., Zhan, L., et al. (2021). clusterProfiler 4.0: A universal enrichment tool for interpreting omics data. Innovation *2*, 100141.

OMTN, Volume *35*

Supplemental information

Effective AAV-mediated gene replacement therapy in retinal organoids modeling AIPL1-associated LCA4

Hali Sai, Bethany Ollington, Farah O. Rezek, Niuzheng Chai, Amelia Lane, Tassos Georgiadis, James Bainbridge, Michel Michaelides, Almudena Sacristan-Reviriego, Pedro R.L. Perdigão, Amy Leung, and Jacqueline van der Spuy

SUPPLEMENTAL INFORMATION

SUPPLEMENTAL TABLES

Table S1. Primary and secondary antibodies used in immunofluorescence (IF) and western blot (WB) analysis.

Antibody	Species	Fluorophore conjugate	Supplier	Cat number	Dilution
AIPL1	Rabbit	-	Produced in-house	N/A	1:250 (IF)
AIPL1	Rabbit	-	V. Ramamurthy lab	N/A	1:1000 (WB)
Cone arrestin	Mouse	-	Millipore	MABN2636	1:250 (IF)
GAPDH	Mouse	-	ProteinTech	60004-1-Ig	1:10,000 (WB)
PDE6A	Rabbit	-	ProteinTech	21200-1-AP	1:1000 (IF) 1:3000 (WB)
PDE6B	Rabbit	-	Thermo Fisher Scientific	PA1-722	1:500 (IF)
Rhodopsin	Mouse	-	Millipore	MABN15	1:1000 (IF)
α-mouse IgG	Donkey	AF488	Thermo Fisher Scientific	A-21202	1:1000 (IF)
α-mouse IgG	Donkey	AF555	Thermo Fisher Scientific	A-32773	1:1000 (IF)
α-mouse IgG	Donkey	AF647	Thermo Fisher Scientific	A-31571	1:1000 (IF)
α-rabbit IgG	Donkey	AF488	Thermo Fisher Scientific	A-21206	1:1000 (IF)
α-rabbit IgG	Donkey	AF555	Thermo Fisher Scientific	A-31572	1:1000 (IF)
α-rabbit IgG	Donkey	AF647	Thermo Fisher Scientific	A-31573	1:1000 (IF)
α-mouse IgG	Goat	HRP	Thermo Fisher Scientific	31430	1:10,000 (WB)
α-rabbit IgG	Goat	HRP	Thermo Fisher Scientific	31461	1:10,000 (WB)

Phalloidin	-	AF488	Invitrogen / Molecular probes	A12379	5 U/mL (IF)

Table S2. Primer sequences for RT-PCR analysis of AIPL1-associated markers.

Marker	Forward	Reverse	Expected product size (bp)
AIPL1	ACCGGATCCCGA GTGATCTT	CGATGATGATGTG CATGGGC	66
CRX	TTTGCCAAGACC CAGTACC	GTTCTTGAACCAA ACCTGAAC	96
GRK1	GAAGCTGAACAA GAAGCGG	GACACGATGAACC TGCTGT	99
PDE6A	TAACGTCCCCAA CACAGAGG	CCACCACATCCTT CCCATTC	116
PDE6B	GACGTGTGGTCT GTGCTGAT	CTTGCCGTGGAGG ATGTAGTC	111
PDE6C	GTCACTAAGAAC CTGCTGGCAACC	AAAGACCTCTTCA TCCTGTTTGG	117
PDE6G	AAGCAGCGACAG ACCAGG	TGTGATGTCTGTT CCCAGGC	105
PDE6H	GAGGCAGACTCG CCAATTC	GTGGCTGAATGCC TCCCA	130

SUPPLEMENTAL FIGURES

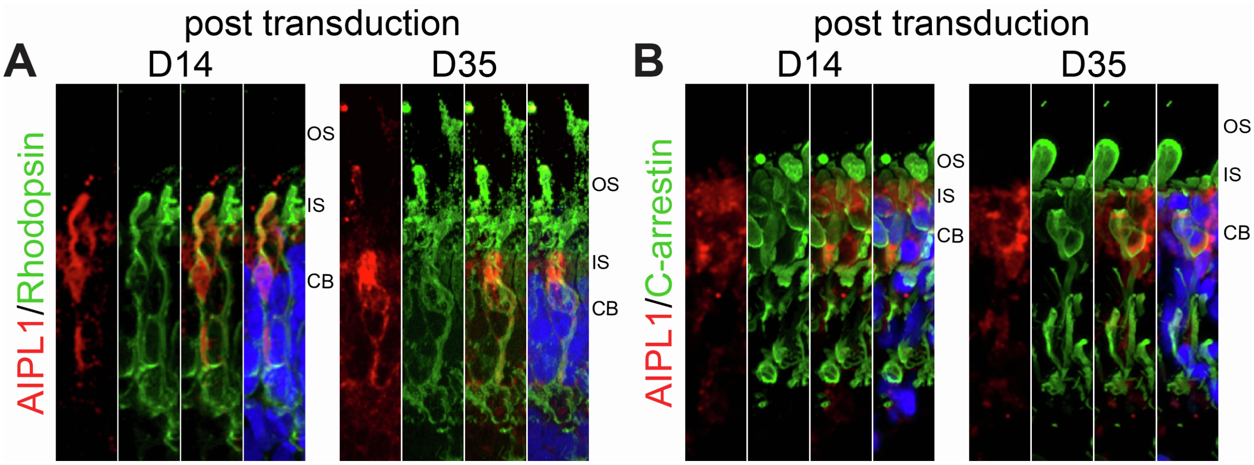

Figure S1: AAV transduction of rod and cone photoreceptors

(A) Expression of AIPL1 (red) and rhodopsin (green) and (B) AIPL1 (red) and cone arrestin (green) in LCA4 patient-derived (LCA4) ROs transduced with AAV7m8.*hRKp.AIPL1* at day 196 of differentiation and analysed 14 days (D14) and 35 days (D35) post transduction.

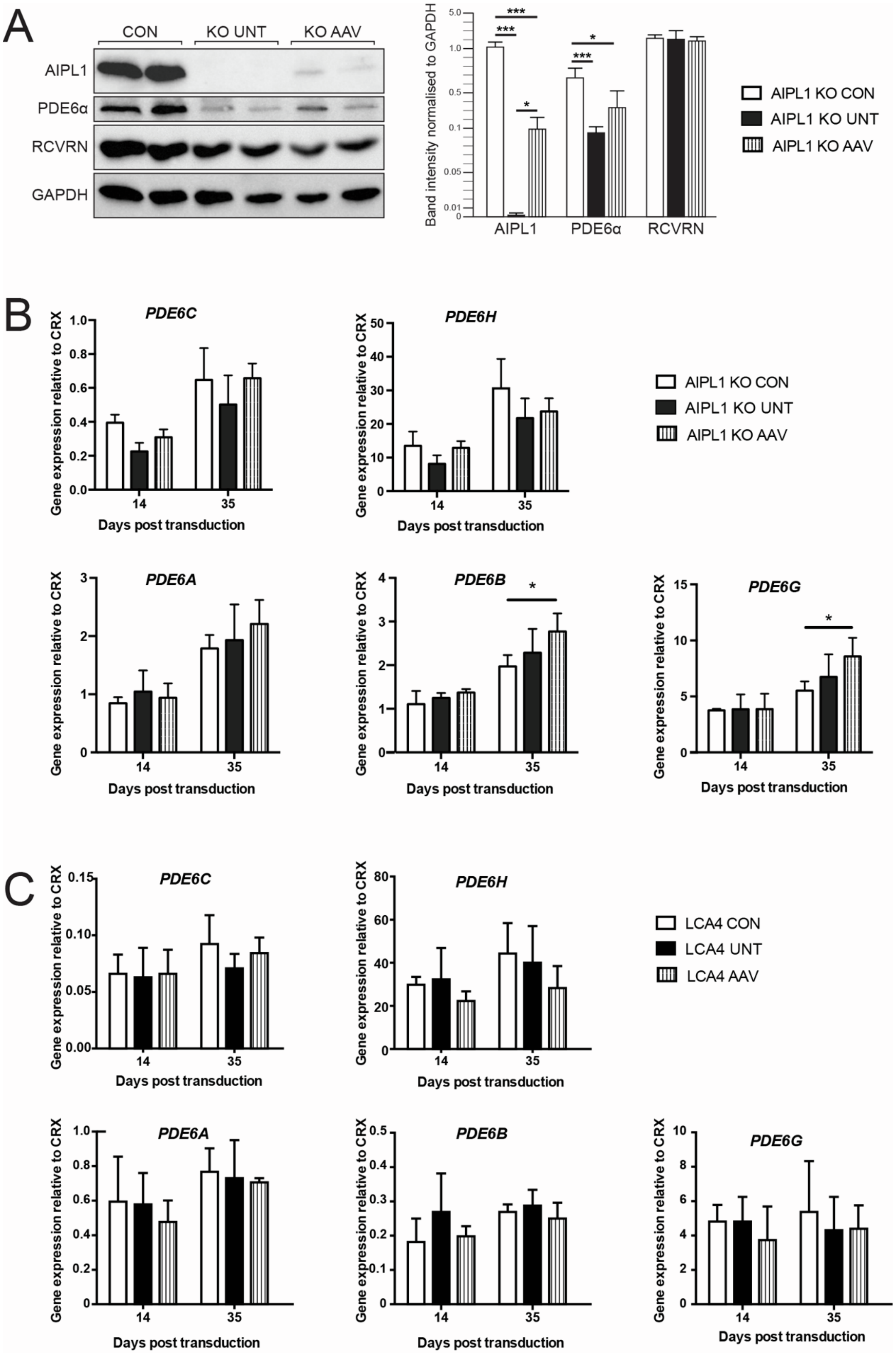
A
CON
KO UNT
KO AAV
AIPL1
PDE6α
RCVRN
GAPDH
Band intensity normalised to GAPDH
AIPL1
PDE6α
RCVRN
AIPL1 KO CON
AIPL1 KO UNT
AIPL1 KO AAV
B
PDE6C
PDE6H
PDE6A
PDE6B
PDE6G
Gene expression relative to CRX
Days post transduction
AIPL1 KO CON
AIPL1 KO UNT
AIPL1 KO AAV
C
PDE6C
PDE6H
PDE6A
PDE6B
PDE6G
Gene expression relative to CRX
Days post transduction
LCA4 CON
LCA4 UNT
LCA4 AAV

Figure S2: Rescue of PDE6 is post-transcriptional

(A) Key expression of LCA4 related proteins following AAV7m8.*hRKp.AIPL1* treatment in the AIPL1 KO model. Western blot of 3-6 pooled ROs per lane from isogenic control (CON), AIPL1 KO untreated (KO UNT) and AIPL1 KO AAV treated (KO AAV) ROs probed for AIPL1, PDE6α, Recoverin (RCVRN) and GAPDH as a loading control, and densitometry calculated relative to GAPDH (n = 2 independent experiments). Statistical significance determined by one-way ANOVA where *, **, *** denote a p value ≤0.05, ≤0.01 and ≤0.005 respectively. Total RNA was isolated from AIPL1 KO ROs (B) and LCA4 patient-derived ROs (C) treated with AAV7m8.*hRKp.AIPL1* at day 196 of differentiation and analysed 14 days (2 weeks) and 35 days (5 weeks) post transduction. The expression of *PDE6C*, *PDE6H*, *PDE6A*, *PDE6B* and *PDE6G* in isogenic control (CON), untreated (UNT) and AAV treated (AAV) ROs was assessed by qPCR relative to the expression of *CRX*. N = 3 individual ROs per sample (with n = 3 technical replicates per RO). Statistical significance determined by one-way ANOVA, where *, **, *** denotes p<0.05, 0.01 and 0.005 respectively.

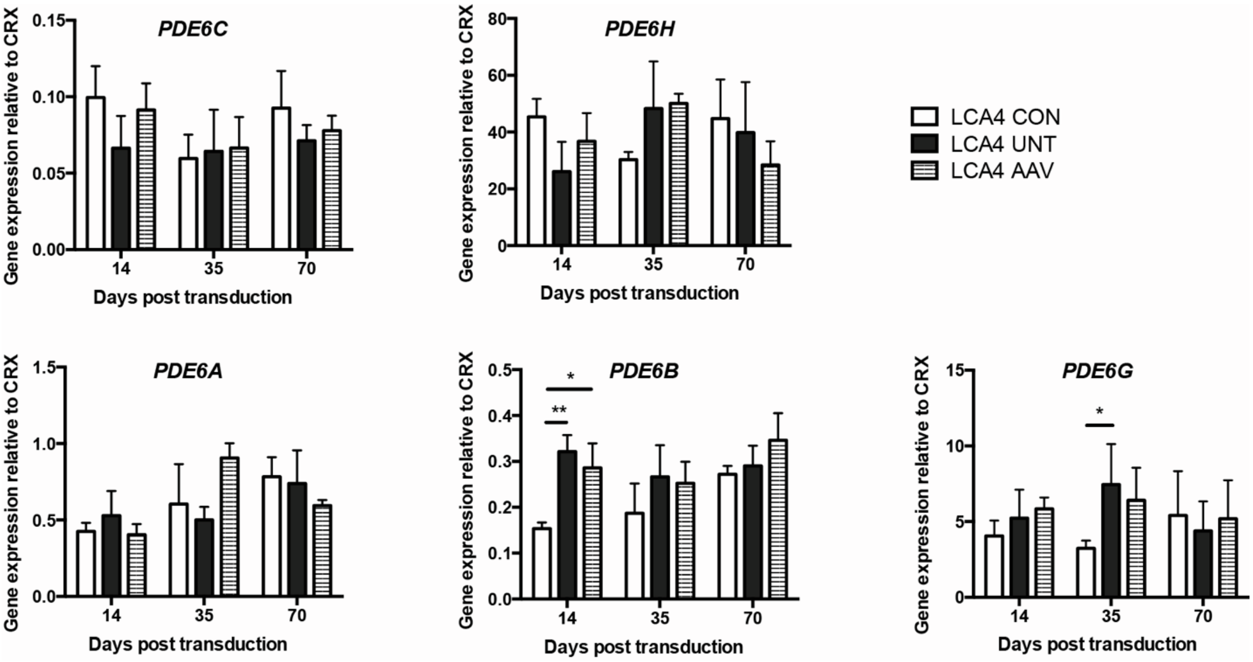

Figure S3: PDE6 transcript levels are unchanged several weeks after AAV treatment

Total RNA was isolated from LCA4 patient-derived ROs treated with AAV7m8.*hRKp.AIPL1* at day 161 of differentiation and analysed 14 days (2 weeks), 35 days (5 weeks) and 70 days (10 weeks) post transduction. The expression of *PDE6C*, *PDE6H, PDE6A*, *PDE6B*, and *PDE6G* in isogenic control (CON), untreated (UNT) and AAV treated (AAV) ROs was assessed by qPCR relative to the expression of *CRX*. N = 3 individual ROs per sample (with n = 3 technical replicates per RO). Statistical significance determined by one-way ANOVA, where *, **, *** denotes p<0.05, 0.01 and 0.005 respectively.

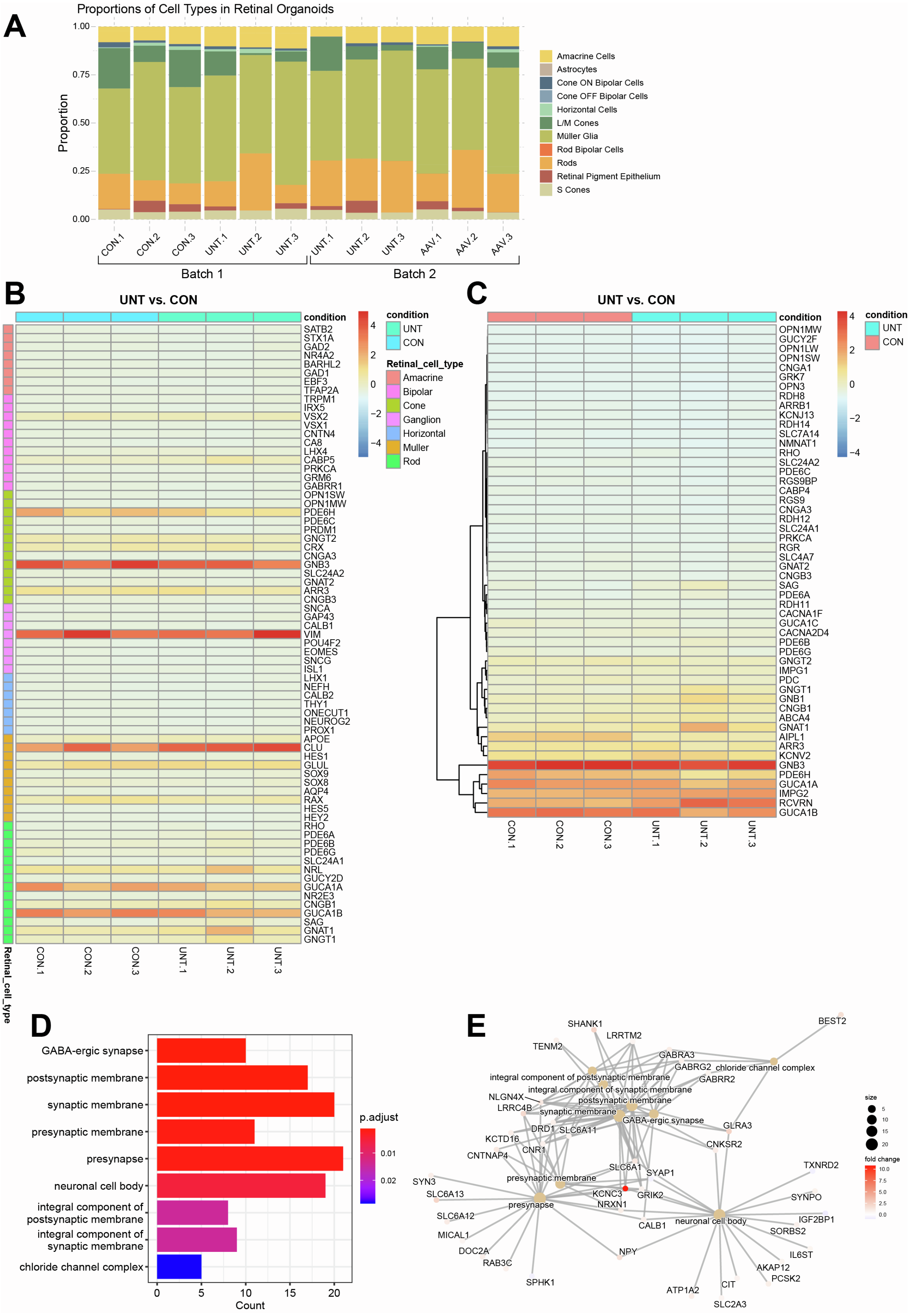
A
Proportions of Cell Types in Retinal Organoids
Proportion
1.00
0.75
0.50
0.25
0.00
CON.1
CON.2
CON.3
UNT.1
UNT.2
UNT.3
UNT.1
UNT.2
UNT.3
AAV.1
AAV.2
AAV.3
Batch 1
Batch 2
Amacrine Cells
Astrocytes
Cone ON Bipolar Cells
Cone OFF Bipolar Cells
Horizontal Cells
L/M Cones
Müller Glia
Rod Bipolar Cells
Rods
Retinal Pigment Epithelium
S Cones
B
UNT vs. CON
condition
UNT
CON
Retinal_cell_type
Amacrine
Bipolar
Cone
Ganglion
Horizontal
Muller
Rod
SATB2
STX1A
GAD2
NR4A2
BARHL2
GAD1
EBF3
TFAP2A
TRPM1
IRX5
VSX2
VSX1
CNTN4
CA8
LHX4
CABP5
PRKCA
GRM6
GABRR1
OPN1SW
OPN1MW
PDE6H
PDE6C
PRDM1
GNGT2
CRX
CNGA3
GNB3
SLC24A2
GNAT2
ARR3
CNGB3
SNCA
GAP43
CALB1
VIM
POU4F2
EOMES
SNCG
ISL1
LHX1
NEFH
CALB2
THY1
ONECUT1
NEUROG2
PROX1
APOE
CLU
HES1
GLUL
SOX9
SOX8
AQP4
RAX
HES5
HEY2
RHO
PDE6A
PDE6B
PDE6G
SLC24A1
NRL
GUCY2D
GUCA1A
NR2E3
CNGB1
GUCA1B
SAG
GNAT1
GNGT1
CON.1
CON.2
CON.3
UNT.1
UNT.2
UNT.3
C
UNT vs. CON
condition
UNT
CON
OPN1MW
GUCY2F
OPN1LW
OPN1SW
CNGA1
GRK7
OPN3
RDH8
ARRB1
KCNJ13
RDH14
SLC7A14
NMNAT1
RHO
SLC24A2
PDE6C
RGS9BP
CABP4
RGS9
CNGA3
RDH12
SLC24A1
PRKCA
RGR
SLC4A7
GNAT2
CNGB3
SAG
PDE6A
RDH11
CACNA1F
GUCA1C
CACNA2D4
PDE6B
PDE6G
GNGT2
IMPG1
PDC
GNGT1
GNB1
CNGB1
ABCA4
GNAT1
AIPL1
ARR3
KCNV2
GNB3
PDE6H
GUCA1A
IMPG2
RCVRN
GUCA1B
CON.1
CON.2
CON.3
UNT.1
UNT.2
UNT.3
D
GABA-ergic synapse
postsynaptic membrane
synaptic membrane
presynaptic membrane
presynapse
neuronal cell body
integral component of postsynaptic membrane
integral component of synaptic membrane
chloride channel complex
p.adjust
0.01
0.02
Count
E
SHANK1
LRRTM2
TENM2
BEST2
GABRA3
GABRG2
chloride channel complex
GABRR2
integral component of postsynaptic membrane
integral component of synaptic membrane
NLGN4X
LRRC4B
postsynaptic membrane
synaptic membrane
GABA-ergic synapse
GLRA3
DRD1
SLC6A11
KCTD16
CNR1
CNTNAP4
CNKSR2
TXNRD2
SLC6A1
SYAP1
SYN3
presynaptic membrane
SLC6A13
KCNC3
GRIK2
SYNPO
NRXN1
presynapse
SLC6A12
CALB1
neuronal cell body
IGF2BP1
SORBS2
MICAL1
DOC2A
NPY
IL6ST
RAB3C
AKAP12
PCSK2
SPHK1
ATP1A2
CIT
SLC2A3
size
fold change

Figure S4: Transcriptomic analysis of LCA4 patient-derived ROs

(A) Estimation of different cell types and proportions in isogenic control ROs (CON), LCA4 patient-derived untreated ROs (UNT) and LCA4 patient-derived AAV treated ROs (AAV) by cell type deconvolution (MuSiC v.1.0.0) of Raw Bulk RNAseq count data.[27] Publicly available single cell RNAseq data from similar-aged retinal organoids was used as a reference for MuSiC.[28] Statistical significance ($p < 0.05$) for individual cell types across conditions within each batch was determined by Kruskal-Wallis tests. (B, C) Heatmaps of normalised count values scaled by column for genes of interest from isogenic control (CON) and untreated LCA4 patient-derived (UNT) ROs. Specific genes of interest for retinal cell types (amacrine cells, bipolar cells, cone photoreceptors, ganglion cells, horizontal cells, Müller glia and rod photoreceptors) and the phototransduction/visual cascade are shown in (B) and (C) respectively. (D) Bar chart and (E) netplot of over-representation analysis of cellular compartments enriched in the significantly differentially expressed gene-set comparing untreated LCA4 patient-derived ROs to isogenic control ROs.

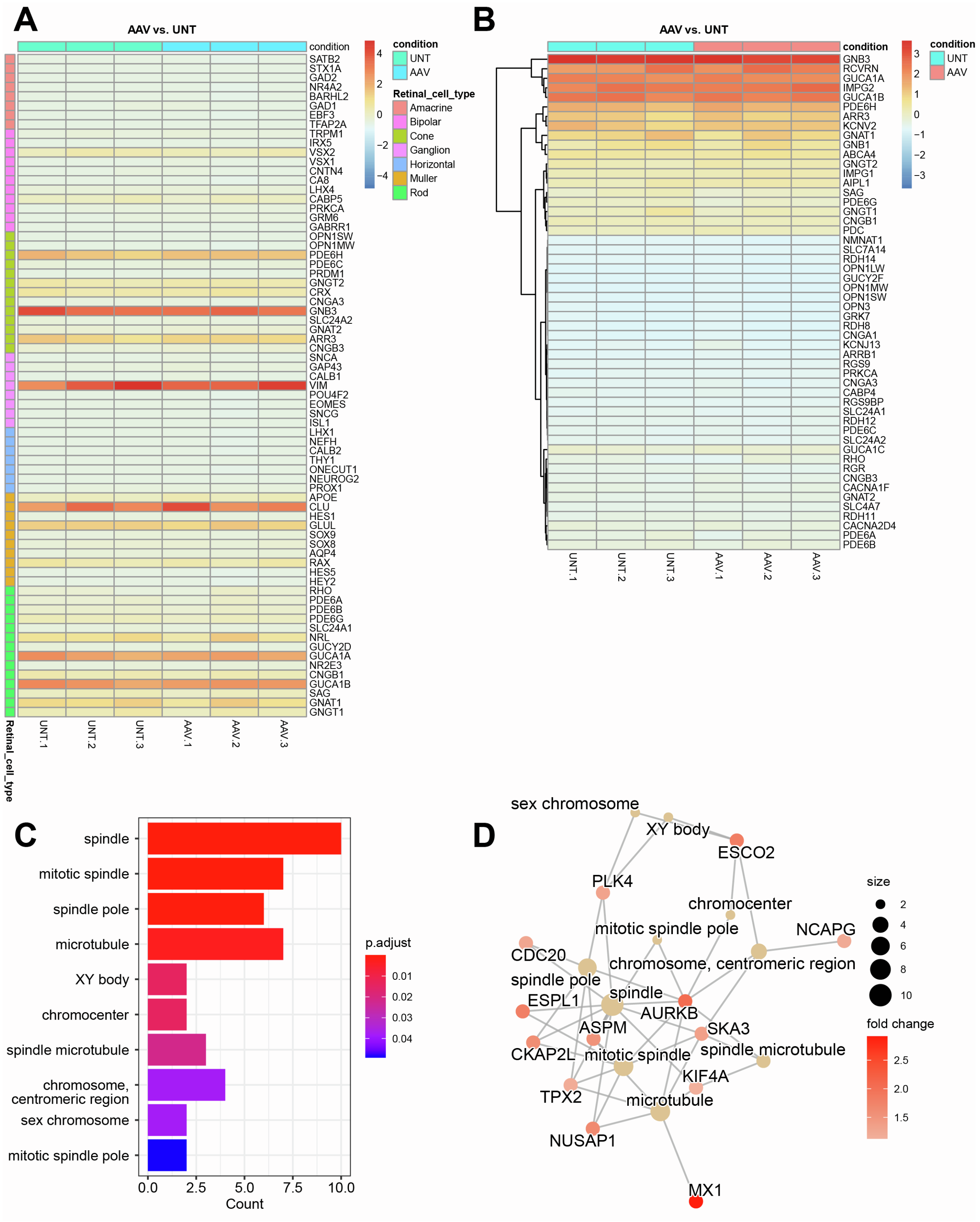
A
AAV vs. UNT
condition
SATB2
STX1A
GAD2
NR4A2
BARHL2
GAD1
EBF3
TFAP2A
TRPM1
IRX5
VSX2
VSX1
CNTN4
CA8
LHX4
CABP5
PRKCA
GRM6
GABRR1
OPN1SW
OPN1MW
PDE6H
PDE6C
PRDM1
GNGT2
CRX
CNGA3
GNB3
SLC24A2
GNAT2
ARR3
CNGB3
SNCA
GAP43
CALB1
VIM
POU4F2
EOMES
SNCG
ISL1
LHX1
NEFH
CALB2
THY1
ONECUT1
NEUROG2
PROX1
APOE
CLU
HES1
GLUL
SOX9
SOX8
AQP4
RAX
HES5
HEY2
RHO
PDE6A
PDE6B
PDE6G
SLC24A1
NRL
GUCY2D
GUCA1A
NR2E3
CNGB1
GUCA1B
SAG
GNAT1
GNGT1
UNT.1
UNT.2
UNT.3
AAV.1
AAV.2
AAV.3
Retinal_cell_type
4
2
0
−2
−4
condition
UNT
AAV
Retinal_cell_type
Amacrine
Bipolar
Cone
Ganglion
Horizontal
Muller
Rod
B
AAV vs. UNT
condition
GNB3
RCVRN
GUCA1A
IMPG2
GUCA1B
PDE6H
ARR3
KCNV2
GNAT1
GNB1
ABCA4
GNGT2
IMPG1
AIPL1
SAG
PDE6G
GNGT1
CNGB1
PDC
NMNAT1
SLC7A14
RDH14
OPN1LW
GUCY2F
OPN1MW
OPN1SW
OPN3
GRK7
RDH8
CNGA1
KCNJ13
ARRB1
RGS9
PRKCA
CNGA3
CABP4
RGS9BP
SLC24A1
RDH12
PDE6C
SLC24A2
GUCA1C
RHO
RGR
CNGB3
CACNA1F
GNAT2
SLC4A7
RDH11
CACNA2D4
PDE6A
PDE6B
UNT.1
UNT.2
UNT.3
AAV.1
AAV.2
AAV.3
3
2
1
0
−1
−2
−3
condition
UNT
AAV
C
spindle
mitotic spindle
spindle pole
microtubule
XY body
chromocenter
spindle microtubule
chromosome, centromeric region
sex chromosome
mitotic spindle pole
0.0
2.5
5.0
7.5
10.0
Count
p.adjust
0.01
0.02
0.03
0.04
D
sex chromosome
XY body
ESCO2
PLK4
chromocenter
mitotic spindle pole
NCAPG
CDC20
chromosome, centromeric region
spindle pole
spindle
ESPL1
AURKB
ASPM
SKA3
CKAP2L
mitotic spindle
spindle microtubule
KIF4A
TPX2
microtubule
NUSAP1
MX1
size
2
4
6
8
10
fold change
2.5
2.0
1.5

Figure S5: Transcriptomic analysis of LCA4 patient-derived ROs following AAV treatment

(A, B) Heatmaps of normalised count values scaled by column for genes of interest from untreated LCA4 patient-derived (UNT) and treated LCA4 patient-derived ROs (AAV). Specific genes of interest for retinal cell types (amacrine cells, bipolar cells, cone photoreceptors, ganglion cells, horizontal cells, Müller glia and rod photoreceptors) and the phototransduction/visual cascade are shown in (A) and (B) respectively. (C) Bar chart and (D) netplot of over-representation analysis of cellular components enriched in the significantly differentially expressed gene-set comparing AAV treated to untreated LCA4 patient-derived ROs.

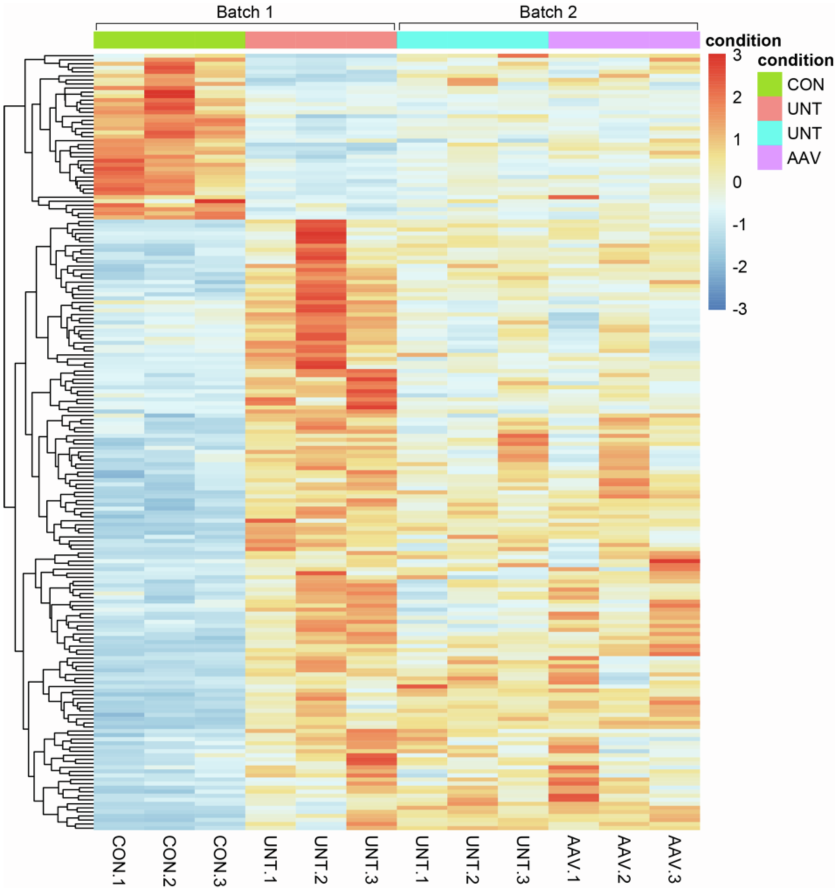

Figure S6: Comparison of gene expression profiles of isogenic control, untreated and AAV treated LCA4 patient ROs.

Heatmap of normalised count values scaled by row for genes of interest from isogenic control (CON), untreated LCA4 patient-derived (UNT) and treated LCA4 patient-derived (AAV) ROs. Genes differentially expressed (padj < 0.05, shrunken LFC > 0.585) between the isogenic control and untreated samples were used to generate the heatmap. Values are represented by red or blue, which indicate relative over- or under-expression of genes, respectively.
